# Exploring the Potential Role of Hydroxytyrosol in Androgenetic Alopecia: An Integrated Bioinformatics and Molecular Simulation Study

**DOI:** 10.3390/ijms27114858

**Published:** 2026-05-28

**Authors:** Leying Zhang, Xiaoyang An, Meihong Xu

**Affiliations:** 1 Department of Nutrition and Food Hygiene, School of Public Health, Beijing Key Laboratory of Toxicological Research and Risk Assessment for Food Safety, Peking University, Beijing 100191, China; leying@stu.pku.edu.cn (L.Z.); 2010306226@stu.pku.edu.cn (X.A.); 2 Institute of Medical Technology, Peking University Health Science Center, Beijing 100191, China

**Keywords:** androgenetic alopecia, hydroxytyrosol, inflammation, bioinformatics analysis, PTGS2, molecular dynamics simulation

## Abstract

Androgenetic alopecia (AGA) is associated with follicular miniaturization, oxidative stress, and microinflammation. Hydroxytyrosol (HT) has antioxidant and anti-inflammatory activities, but its mechanisms in AGA remain unclear. Differentially expressed genes (DEGs) from GSE212301 were intersected with predicted HT targets to identify candidate targets. Enrichment analysis, PPI-based hub gene screening, molecular docking, and molecular dynamics (MD) simulations were performed. A total of 506 DEGs and 24 HT-related AGA candidate targets were identified. These targets were enriched in inflammation- and oxidative stress-related pathways, including MAPK, NF-κB, TNF, and HIF-1 signaling. *HMOX1*, *PTGS2*, *FOS*, and *JUN* were identified as hub genes. Docking showed favorable binding between HT and these targets, particularly PTGS2. The 50 ns MD simulation supported the relative stability of the HT–PTGS2 complex. HT may modulate AGA-related inflammatory and oxidative stress networks, particularly through HMOX1 and PTGS2, providing a basis for further experimental validation.

## 1. Introduction

Androgenetic alopecia (AGA) is a common chronic non-scarring form of hair loss, also known as pattern baldness, which primarily affects specific areas of the scalp, such as the vertex and frontal regions in men [1,2]. The hallmark pathological feature of this condition is progressive hair follicle miniaturization. As the disease progresses, the hair follicles gradually undergo atrophy and decrease in number, and may become non-functional [3]. AGA has become a global issue. Epidemiological studies indicate that the global prevalence of AGA is approximately 24.88% in men and 5.06% in women [4]. This condition significantly affects patients’ quality of life.

Current evidence suggests that genetic predisposition, abnormal levels of dihydrotestosterone (DHT), environmental exposure, and specific chemical factors are major contributors to AGA [5]. The binding of DHT to androgen receptors and subsequent activation is considered a key mechanism driving hair follicle miniaturization [6]. Current therapeutic strategies primarily target androgen signaling pathways, including androgen receptors and 5α-reductase, with commonly used drugs such as minoxidil and finasteride [7]. However, these treatments are often limited by variable efficacy and adverse effects, highlighting the need for alternative strategies, including nutritional interventions.

Emerging evidence indicates that microinflammatory responses within hair follicles play an important role in the pathogenesis of AGA. Unlike classical acute inflammation, AGA is characterized by a chronic, low-grade microinflammatory process. However, the underlying mechanisms remain incompletely understood. The initiation of progressive perifollicular fibrosis has been associated with T-cell infiltration in the follicular stem cell epithelium [8]. Infiltration of inflammatory cells induces the secretion of cytokines, disrupts the normal hair growth cycle, and contributes to hair follicle miniaturization [9,10,11]. Long-term continuous infiltration of inflammatory cells leads to fibrosis in the surrounding tissue of the follicles, reducing local blood supply and resulting in insufficient oxygen supply. Hypoxic conditions further promote the accumulation of reactive oxygen species (ROS), which can disrupt cellular homeostasis. Short-term accumulation of ROS can stimulate hair follicle proliferation, but in androgenetic alopecia, the dermal papilla cells (DPCs) of the alopecia show a long-term increase in ROS levels, which leads to the DPCs being in a more pro-apoptotic state, thereby preventing hair growth [12,13]. Therefore, targeting inflammation and oxidative stress represents a promising therapeutic strategy for AGA.

The Mediterranean diet, characterized by high intake of olive oil and plant-derived antioxidants, has been associated with anti-inflammatory and antioxidative effects. It may be able to regulate AGA. Clinical studies have confirmed that the Mediterranean diet, with olive oil as an important component, can reduce the proportion of resting hair and the proportion of miniaturized hair in the growth phase, resist oxidative stress, and regulate inflammation, thereby reducing the risk of hair loss [14,15,16]. Hydroxytyrosol (HT), a major polyphenol derived from olive oil, is recognized as a potent natural antioxidant with anti-inflammatory properties [17]. Previous studies have demonstrated that HT can inhibit inflammatory signaling pathways and modulate cellular stress responses [18]. Serra et al. reported that HT has the effect of inhibiting COX-2 expression by blocking NF-κB activation, suggesting its anti-inflammatory potential [19]. HT can also penetrate the cell membrane and neutralize ROS, demonstrating a strong scavenging activity against ROS [20]. Given that the biological functions of HT align with key pathogenic mechanisms of AGA, it may have potential as a candidate intervention.

Despite its known biological activities, direct evidence supporting the role of HT in AGA remains limited, and the underlying molecular mechanisms remain to be elucidated. Therefore, this study aimed to systematically explore the potential molecular targets and pathways of HT in AGA using an integrated bioinformatics and molecular simulation approach.

## 2. Results

### 2.1. Differentially Expressed Genes Analysis

**Figure 1:**
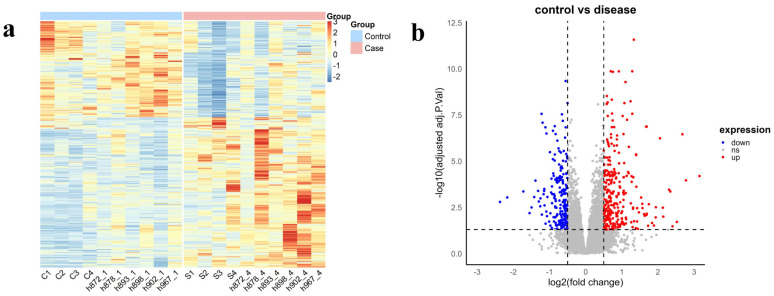
(**a**) Heatmap of differentially expressed genes from GSE212301. Samples C1–C4 and h872_1–h967_1 belong to the control group, whereas samples S1–S4 and h872_4–h967_4 belong to the case group. (**b**) Volcano plot of differentially expressed genes from GSE212301.

The GSE212301 dataset was analyzed to identify DEGs between affected frontal hair follicles and unaffected occipital follicles from AGA patients. A total of 506 DEGs were identified, including 308 upregulated and 198 downregulated genes (Table S1). The overall expression patterns were visualized using heatmaps and volcano plots, demonstrating clear separation between the two groups (Figure 1a,b).

### 2.2. KEGG and GO Enrichment Analysis

**Figure 2:**
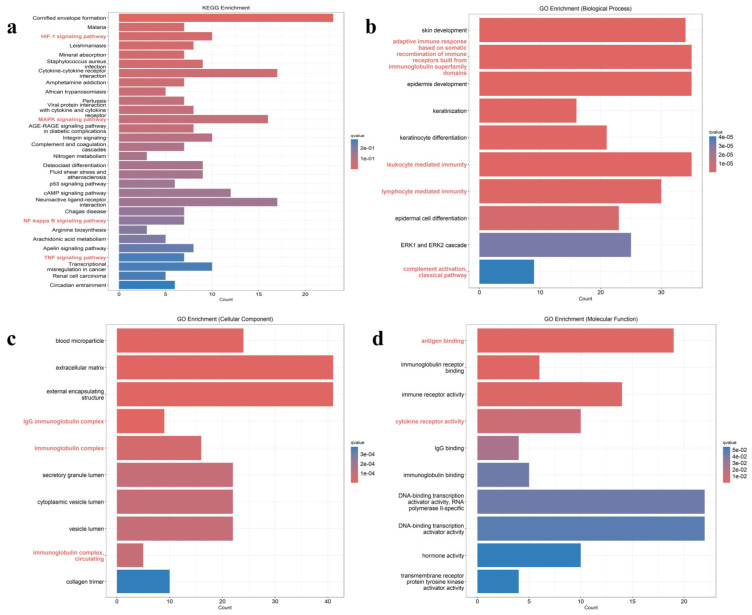
(**a**) KEGG analysis of DEGs. (**b**) The GO analysis of DEGs (biological processes). (**c**) GO cellular component analysis. (**d**) GO molecular function analysis. Red bold labels indicate the key significantly enriched pathways/functions highlighted in this study.

To explore the biological functions of DEGs, GO and KEGG enrichment analyses were performed. KEGG analysis showed that DEGs were significantly enriched in pathways related to inflammation and stress responses, including the MAPK signaling pathway, NF-κB signaling pathway, TNF signaling pathway, HIF-1 signaling pathway, and cytokine–cytokine receptor interaction (Figure 2a).

GO enrichment analysis further indicated that DEGs were primarily involved in immune-related biological processes, such as adaptive immune response, lymphocyte-mediated immunity, and leukocyte-mediated immunity (Figure 2b). At the cellular component level, enrichment was observed in immunoglobulin complexes, while molecular function analysis highlighted antigen-binding and cytokine receptor activity (Figure 2c,d).

### 2.3. Identification and Enrichment Analysis of HT-Related AGA Candidate Targets

**Figure 3:**
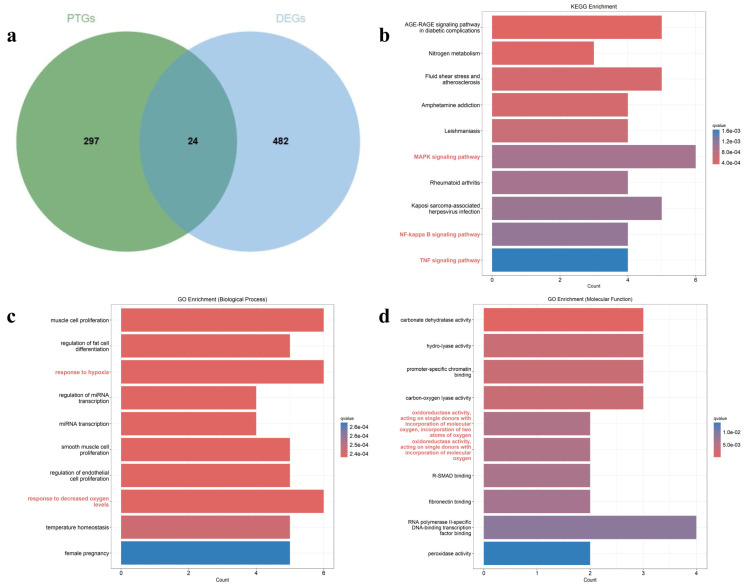
(**a**) Venn diagram showing the intersection between AGA-related DEGs and predicted HT target genes. (**b**) KEGG analysis of DEPTGs. (**c**) The GO analysis of DEPTGs (biological processes). (**d**) The GO analysis of DEPTGs (molecular functions). Red bold labels indicate the key significantly enriched pathways/functions highlighted in this study.

A total of 321 potential targets of HT were predicted using multiple databases. By intersecting these targets with DEGs, 24 candidate genes were identified as potential targets of HT in AGA (Figure 3a). These genes included *HMOX1*, *PTGS2*, *FOS*, *JUN*, *VEGFA*, *ICAM1*, and others, suggesting potential involvement in multiple biological processes.

GO and KEGG enrichment analyses were performed on the 24 candidate genes. KEGG analysis revealed significant enrichment in pathways associated with inflammation and stress regulation, including the MAPK, NF-κB, and TNF signaling pathways (Figure 3b). GO analysis indicated enrichment in biological processes such as response to hypoxia and oxidative stress, as well as molecular functions related to oxidoreductase activity (Figure 3c,d).

### 2.4. PPI Network and Hub Gene Results

**Figure 4:**
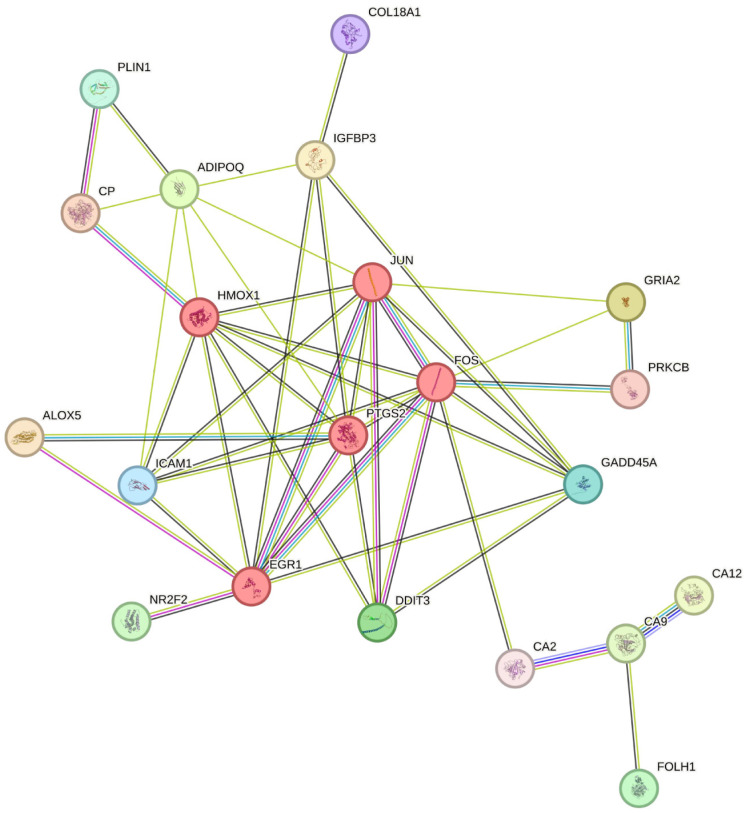
PPI analysis was conducted on the 24 DEPTGs using the STRING database and visualized in Cytoscape (v3.10.4). A confidence score threshold of ≥0.4 was applied, and isolated nodes were removed from the network. The genes highlighted in red represent the top five hub genes. Different edge colors indicate various evidence sources supporting the interactions, including text mining (light green), experimentally determined (pink), from curated databases (light blue), co-expression (black), gene neighborhood (green), gene fusion (red), and gene co-occurrence (dark blue).

A PPI network was constructed for the 24 candidate genes using the STRING database and visualized in Cytoscape (Figure 4). After removing isolated nodes, the network was further analyzed to identify key genes.

Using multiple topological algorithms (MCC, MNC, Degree, and Closeness), 10 hub genes were identified. Among them, *HMOX1*, *PTGS2*, *FOS*, *JUN*, and *EGR1* ranked highest based on integrated scoring (Table 1).

### 2.5. Molecular Docking Analysis

**Figure 5:**
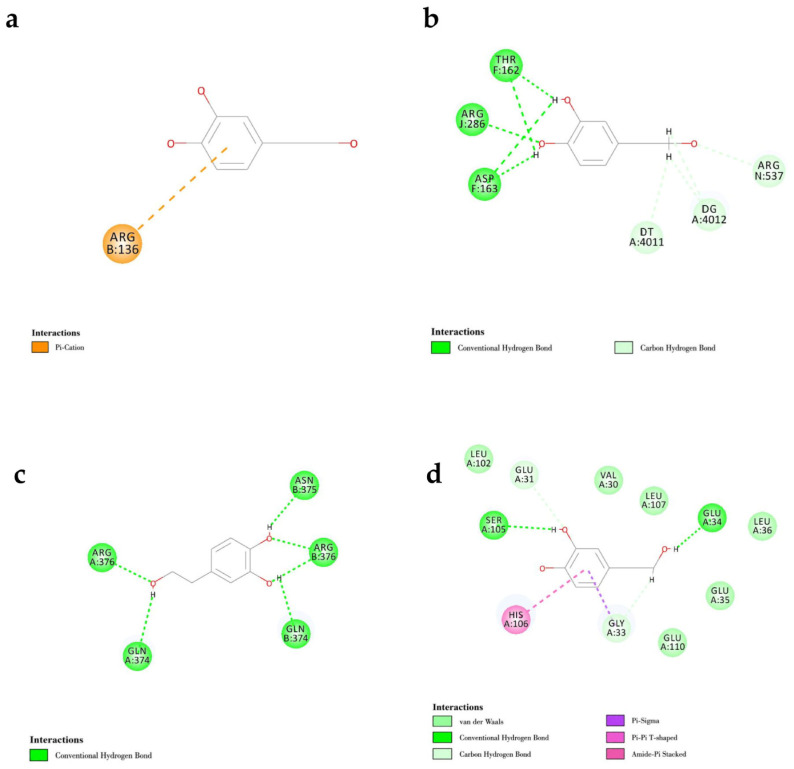
(**a**) Visualization of molecular docking sites between HMOX1 and HT. (**b**) Visualization of molecular docking sites between FOS and HT. (**c**) The visualization of molecular docking sites between PTGS2 and HT. (**d**) The visualization of molecular docking sites between JUN and HT. Green dashed lines indicate hydrogen bonds. Pink dashed lines indicate Pi-Pi T-shaped interactions, while purple dashed lines indicate Pi-Sigma interactions.

Molecular docking was performed to evaluate the binding interactions between HT and selected hub proteins (HMOX1, PTGS2, FOS, and JUN). The docking results demonstrated favorable binding affinities between HT and all four targets. Among these, the HT–PTGS2 complex showed the lowest interaction energy, indicating a relatively stronger binding affinity compared to other targets. Multiple hydrogen bonds and hydrophobic interactions were observed at the binding sites (Figure 5a–d). The binding energy of HMOX1 and HT (-CDOCKER_INTERACTION_ENERGY) was −20.0447 kcal/mol, with an interaction mode of Pi_Cation. The binding energy of FOS and HT was −23.1523 kcal/mol, forming 5 regular hydrogen bonds. The binding energy of PTGS2 and HT was −28.635 kcal/mol, with 6 regular hydrogen bonds playing a role. And the binding energy of JUN and HT was −24.0616 kcal/mol, forming 2 regular hydrogen bonds.

### 2.6. Molecular Dynamics Simulation

**Figure 6:**
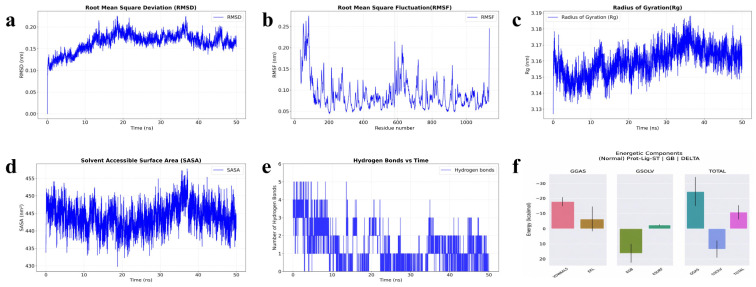
Molecular dynamics simulation analysis of the HT–PTGS2 complex. (**a**) RMSD. (**b**) RMSF. (**c**) Rg. (**d**) SASA. (**e**) Hydrogen bond count. (**f**) Total energy.

To further assess the stability of the HT–PTGS2 complex, a 50 ns molecular dynamics (MD) simulation was conducted. The root mean square deviation (RMSD) values stabilized after approximately 10 ns, fluctuating within a narrow range (~0.15–0.22 nm), indicating conformational stability of the complex (Figure 6a). Root mean square fluctuation (RMSF) analysis showed limited residue fluctuations, with most values below 0.16 nm (Figure 6b). The radius of gyration (Rg) remained stable (~3.14–3.18 nm), suggesting maintenance of structural compactness (Figure 6c). Similarly, the solvent-accessible surface area (SASA) exhibited minor fluctuations, indicating stable solvent exposure (Figure 6d). The number of hydrogen bonds between HT and PTGS2 fluctuated between 0 and 5, with an average of 1–2 bonds during the simulation (Figure 6e). Although fluctuations in total energy were observed, these are commonly associated with solvent and ion dynamics in explicit systems. Overall, the structural parameters support the relative stability of the HT–PTGS2 complex during the 50 ns simulation. Binding free energy analysis further indicated favorable interactions, with contributions from van der Waals and electrostatic interactions.

## 3. Discussion

AGA is increasingly recognized as a multifactorial disorder that extends beyond androgen dependence, involving a complex interplay of oxidative stress, chronic microinflammation, and follicular microenvironment dysfunction [21]. In the present study, we applied an integrated bioinformatics and molecular simulation approach to systematically explore the potential molecular mechanisms of HT in AGA. Our analysis revealed that AGA-related differentially expressed genes were significantly enriched in pathways associated with inflammation and stress responses, including the MAPK, NF-κB, TNF, and HIF-1 signaling pathways. These pathways are highly interconnected and are known to contribute to the establishment of a pro-inflammatory and oxidative microenvironment [20,22,23,24]. Reactive oxygen species (ROS) not only directly impair dermal papilla cells but also activate NF-κB signaling, thereby promoting the production of inflammatory cytokines and forming a self-amplifying cycle [20,23]. These findings support the concept that AGA progression is driven by a coupled network of oxidative stress and inflammation.

By integrating disease-associated genes with predicted HT targets, we identified 24 candidate genes and further screened key hub genes, including *HMOX1*, *PTGS2*, *FOS*, and *JUN*. These genes are central regulators of stress response and inflammatory signaling, suggesting that HT may exert its effects through a multi-target regulatory pattern. Among these, HMOX1 represents a critical antioxidant defense component. HMOX1 catalyzes heme degradation to produce biliverdin and bilirubin, both of which exhibit strong antioxidant properties [25]. Previous studies have shown that HT can upregulate HMOX1 expression, thereby enhancing endogenous antioxidant capacity [20,26,27]. In the context of AGA, where sustained oxidative stress contributes to DPC apoptosis and follicular regression, activation of the HMOX1 axis may help restore redox balance and improve the follicular microenvironment [28].

Another important finding is the potential interaction between HT and PTGS2. Molecular docking and MD simulations indicated favorable binding between HT and PTGS2, with structural parameters supporting the stability of the complex. Although fluctuations in total energy were observed during the MD simulation, such variations are commonly associated with solvent and ion dynamics in explicit systems and do not necessarily indicate instability. Notably, RMSD, Rg, and hydrogen bond analyses remained relatively stable, supporting the conformational stability of the HT–PTGS2 complex. PTGS2 is a key enzyme in prostaglandin synthesis and plays an important role in inflammatory amplification. In AGA, PTGS2-derived prostaglandins, particularly PGD_2_, have been reported to inhibit hair growth and promote follicular miniaturization [29]. Moreover, PTGS2 is involved in a PTGS2/PGE_2_/NF-κB positive feedback loop, which can further enhance inflammatory signaling and ROS production [30,31,32]. Therefore, modulation of PTGS2 by HT may contribute to the attenuation of both inflammation and oxidative stress, potentially disrupting this pathogenic feedback loop.

Interestingly, our study showed decreased PTGS2 expression in AGA samples, which appears inconsistent with previous reports describing PTGS2 upregulation under androgen stimulation [30]. This discrepancy may be explained by differences in sample selection, particularly with respect to hair follicle cycling stages. Although the dataset used in this study consisted of anagen hair follicles, follicles in AGA-affected regions may exhibit functional impairment or premature transition toward catagen-like states. Given that PTGS2 expression is dynamically regulated during the hair cycle [33,34], such stage-dependent variation may account for the observed expression pattern. This highlights the importance of considering biological heterogeneity in transcriptomic analyses.

In addition to inflammation and oxidative stress, other cellular processes, such as apoptosis and autophagy, have also been implicated in AGA pathogenesis. Previous studies have demonstrated that HT can modulate autophagy and protect against oxidative stress-induced cellular damage [14]. Although these processes were not directly investigated in the present study, they may represent additional mechanisms through which HT exerts potential protective effects.

Natural polyphenols such as resveratrol and curcumin have also been reported to interact with COX-2/PTGS2 in molecular docking and molecular dynamics studies, suggesting that modulation of inflammatory enzymes may be a shared feature of antioxidant polyphenols [35,36,37,38,39]. Compared with resveratrol (228.25 g/mol) and curcumin (368.38 g/mol), HT has a relatively lower molecular weight (154.16 g/mol) and a simple catechol structure, which may be favorable for absorption and tissue accessibility. However, docking scores and binding energies obtained from different studies are highly dependent on protein structures, docking algorithms, scoring functions, force fields, and simulation settings. Therefore, direct quantitative comparison across studies should be interpreted cautiously. The present study does not conclude that HT is superior to other polyphenols. Instead, our findings support HT as a promising candidate that warrants future head-to-head comparison with other antioxidant polyphenols under standardized computational and experimental conditions.

Several limitations should be acknowledged. First, this study was primarily based on computational analyses, and the predicted targets and pathways lack direct experimental validation. Second, the transcriptomic analysis was based on the GSE212301 dataset, which contains 10 paired hair follicle samples from AGA patients. Although the intra-individual paired design, comparing affected frontal and unaffected occipital hair follicles from the same patients, helps reduce inter-individual confounding and improves the reliability of DEG identification within this dataset, the relatively small sample size may still limit the robustness and generalizability of the findings. Therefore, larger independent cohorts and multi-center clinical samples are needed to validate the candidate targets and pathways identified in this study. Third, the complex regulatory relationships among the identified pathways were not fully elucidated. Fourth, although the 50 ns MD simulation provided preliminary support for the relative stability of the HT–PTGS2 complex based on RMSD, RMSF, Rg, SASA, hydrogen bond, total energy, and binding free energy analyses, longer simulations and replicate runs would provide additional evidence for dynamic stability.

Future studies should focus on validating these findings using in vitro models, such as human dermal papilla cells, and in vivo models of androgen-induced alopecia. In addition, integrating multi-omics data and functional assays will be important for further elucidating the molecular network underlying HT-mediated effects.

## 4. Materials and Methods

### 4.1. Data Acquisition and Differential Expression Analysis

The gene expression dataset GSE212301 associated with AGA was obtained from the Gene Expression Omnibus (GEO) database (https://www.ncbi.nlm.nih.gov/geo/, accessed on 18 November 2025) [40]. This dataset contains 10 pairs of hair follicle samples from AGA patients, including affected frontal and unaffected occipital regions, generated on the GPL16791 (Illumina HiSeq 2500, Illumina, San Diego, CA, USA) platform.

**Figure 7:**
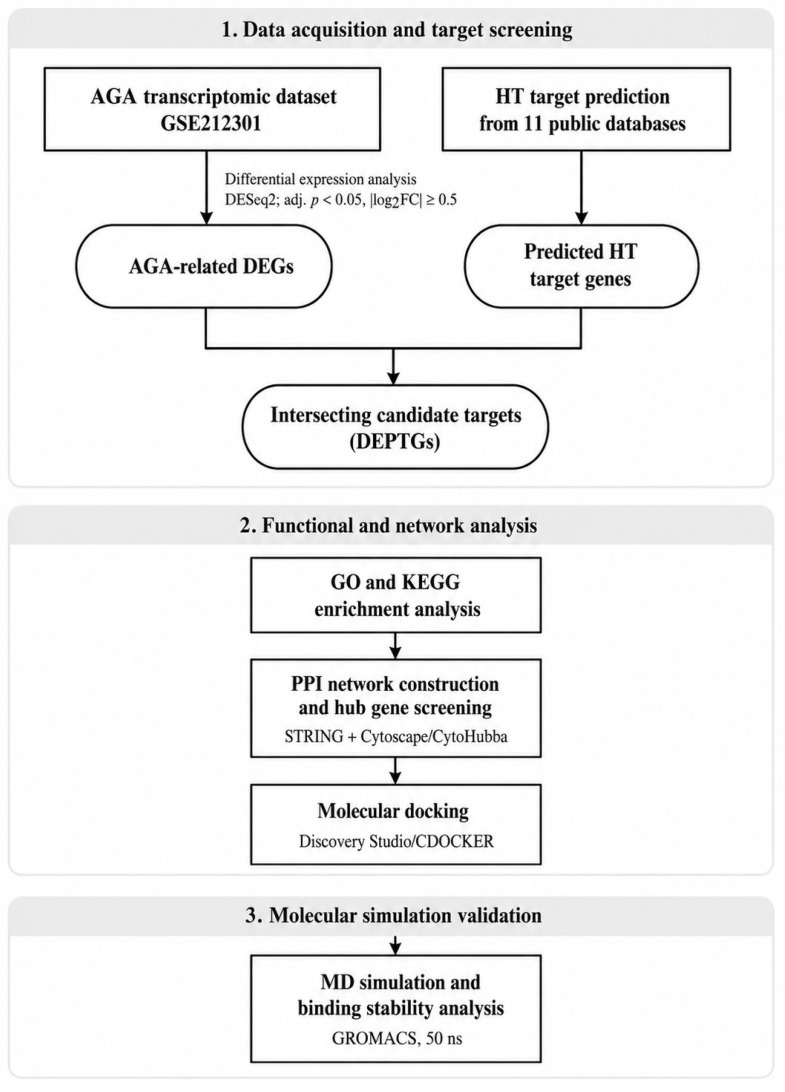
Workflow of the integrated bioinformatics and molecular simulation analysis. AGA-related DEGs were identified from GSE212301, and predicted HT target genes were obtained from 11 public databases. Intersecting candidate targets were subjected to GO/KEGG enrichment, PPI analysis, molecular docking, and MD simulation.

Paired differential gene expression analysis was performed using the DESeq2 package (v1.49.3) [41] in R software (v4.5.1). Genes with low expression levels (counts < 10) were excluded. Differentially expressed genes (DEGs) were identified using the thresholds of adjusted *p*-value (P.adj) < 0.05 and |log2FC| ≥ 0.5 [42]. Visualization of DEGs was conducted using the ggplot2 (v4.0.0) and pheatmap packages (v1.0.13). The methodological flowchart of the study is shown in Figure 7.

### 4.2. Prediction of HT Targets

Potential structural targets of HT were predicted using the following databases: SwissTargetPrediction database (http://swisstargetprediction.ch/, accessed on 1 December 2025) [43], PharmMapper database (https://lilab-ecust.cn/pharmmapper/, accessed on 1 December 2025) [44], BindingDB database (https://www.bindingdb.org/, accessed on 1 December 2025) [45], STITCH (http://stitch.embl.de/, accessed on 1 December 2025) [46], Comparative Toxicogenomics Database (CTD; http://ctdbase.org/, accessed on 1 December 2025) [47], Drug–Gene Interaction Database (DGIdb; http://www.dgidb.org/, accessed on 1 December 2025) [48], Traditional Chinese Medicine Systems Pharmacology Database (TCMSP; http://www.tcmsp-e.com, accessed on 1 December 2025) [49], GeneCards database (https://www.genecards.org/, accessed on 1 December 2025) [50], ChEMBL database (https://www.ebi.ac.uk/chembl/, accessed on 1 December 2025) [51], Similarity Ensemble Approach database (SEA, https://sea.bkslab.org/, accessed on 1 December 2025) [52], and TargetNet database (http://targetnet.scbdd.com, accessed on 1 December 2025) [53]. All target genes were standardized using official gene symbols. The intersection between HT targets and AGA-related DEGs was identified as candidate targets and visualized using the Jvenn online tool (https://jvenn.toulouse.inra.fr/app/example.html, accessed on 3 December 2025) [54].

### 4.3. KEGG and GO Enrichment Analyses

Gene Ontology (GO) and Kyoto Encyclopedia of Genes and Genomes (KEGG) enrichment analyses for DEGs were conducted using the clusterProfiler package (v4.17.0) [55] under the org. Hs.eg.db environment (v3.21.0) [56]. Meanwhile, KEGG and GO analyses were performed on the differentially expressed potential target genes (DEPTGs). A *p*-value < 0.05 was considered statistically significant. The top 30 categories from the KEGG enrichment analysis and the top 10 categories from the GO enrichment analysis were visualized.

### 4.4. Protein–Protein Interaction (PPI) Network Construction and Hub Gene Screening

A PPI network of candidate targets was constructed using the STRING database (https://string-db.org/, accessed on 1 December 2025) [57], with a confidence score ≥ 0.4. The network was visualized and analyzed using Cytoscape software (v3.10.4).

Hub genes were identified using the CytoHubba plugin based on four algorithms: maximal clique centrality (MCC), maximum neighborhood component (MNC), degree, and closeness centrality. Genes consistently ranked highly across these methods were selected as hub genes, and the top 10 genes with the highest comprehensive scores were selected as hub genes.

### 4.5. Molecular Docking

The 3D structure of HT was obtained from the PubChem database. (https://pubchem.ncbi.nlm.nih.gov/, accessed on 21 December 2025) [58]. Protein structures of selected hub targets were retrieved from the UniProt (https://www.uniprot.org/uniprotkb, accessed on 23 December 2025) [59] and RCSB Protein Data Bank (RCSB PDB, https://www.rcsb.org/, accessed on 23 December 2025) [60].

Molecular docking was performed using Discovery Studio 2019. The CDOCKER algorithm was applied to calculate binding modes and interaction energies, including the CDOCKER interaction energy. Docking results were visualized to analyze binding interactions.

### 4.6. Molecular Dynamics (MD) Simulation

MD simulation was performed using the GROMACS software (version 2023.4_conda_forge). The protein was parameterized using the Amber force field (Amber14SB), and HT added the Generation Amber force field (GAFF) using acpype (version 2023.10.27). The protein–ligand complex was placed in a cubic simulation box and solvated using the TIP3P water model. Counterions were added to neutralize the system, electrically neutralized by adding 165 sodium ions and 171 chloride ions. Energy minimization was performed using the Steepest Descent algorithm, followed by temperature (NVT) equilibrium using the V-rescale method (tau_t = 0.1, ref_t = 300 K) and pressure (NPT) equilibrium using the C-rescale method (tau_p = 2.0) [61]. A 50 ns production simulation was conducted. System stability was evaluated using root mean square deviation (RMSD), root mean square fluctuation (RMSF), radius of gyration (Rg), solvent-accessible surface area (SASA), hydrogen bond analysis, and total energy. Binding free energy was calculated using the MM/GBSA method.

## 5. Conclusions

This study suggests that HT may exert potential regulatory effects in AGA by modulating inflammation- and oxidative stress-related signaling networks, particularly the MAPK, NF-κB and TNF signaling pathways. HMOX1 and PTGS2 were recognized as key candidate nodes that may play important roles in the protective effects of HT. Collectively, these findings provide a theoretical basis for further experimental validation and highlight the potential of HT as a promising nutritional candidate for AGA intervention.

## Figures and Tables

**Table 1:** Top 10 hub genes and their pathways related to AGA.

Hub Genes	Pathway/Interactions Related to AGA
*FOS*	MAPK, p53, HIF-1α
*JUN*	TNF, MAPK, Wnt, HIF-1α
*HMOX1*	HIF-1α, NF-κB
*PTGS2*	NF-κB, Wnt
*EGR1*	NF-κB
*ICAM1*	p53
*GADD45A*	p53, MAPK
*ADIPOQ*	AMPK
*DDIT3*	MAPK
*IGFBP3*	Direct p53 effectors

The genes in the table are sorted from high to low according to their importance.

## Data Availability

No new data were created or analyzed in this study. Data sharing is not applicable to this article. The data used in this study were obtained from the GEO database under accession number GSE212301 and are publicly available at: https://www.ncbi.nlm.nih.gov/geo/query/acc.cgi?acc=GSE212301 (accessed on 8 May 2026).

## Supplementary Materials

The following supporting information can be downloaded at: https://www.mdpi.com/article/10.3390/ijms27114858/s1.

## Abbreviations

The following abbreviations are used in this manuscript:

AGA	Androgenetic Alopecia
COX-2	Cyclooxygenase-2
CTD	Comparative Toxicogenomics Database
DEGs	differentially expressed genes
DEPTGs	differentially expressed potential target genes
DGIdb	Drug–Gene Interaction Database
DHT	dihydrotestosterone
DPCs	dermal papilla cells
FOS	Fos proto-oncogene
GAFF	Generation Amber force field
GEO	Gene Expression Omnibus
GO	Gene Ontology
HF	hair follicle
HIF-1	Hypoxia-inducible factor-1
HMOX1	Heme Oxygenase 1
HT	hydroxytyrosol
ICAM1	Intercellular Adhesion Molecule 1
JUN	Jun proto-oncogene
KEGG	Kyoto Encyclopedia of Genes and Genomes
log2FC	log2 fold change
MAPK	Mitogen-Activated Protein Kinase
MCC	maximal clique centrality
MD	molecular dynamics
MNC	maximum neighborhood component
NF-κB	nuclear factor kappa-light-chain-enhancer of activated B cells
NPT	constant pressure and temperature ensemble
NVT	constant volume and temperature ensemble
PGD_2_	Prostaglandin D_2_
PGE_2_	Prostaglandin E_2_
PPI	protein–protein interaction
PTGs	predicted target genes
PTGS2	Prostaglandin-endoperoxide synthase 2
RCSB PDB	RCSB Protein Data Bank
Rg	radius of gyration
RMSD	root mean square deviation
RMSF	root mean square fluctuation
ROS	reactive oxygen species
SASA	solvent accessible surface area
SEA	Similarity Ensemble Approach
TCMSP	Traditional Chinese Medicine Systems Pharmacology Database
TIP3P	Transferable Intermolecular Potential 3-Point
TNF	tumor necrosis factor
VEGFA	Vascular endothelial growth factor A

## References

[1] Chen S., Zheng D., Wang H. (2025). Research progress on the pathogenesis of androgenetic alopecia. Eur. J. Dermatol..

[2] Stamatas G.N., Wu J., Pappas A., Mirmirani P., McCormick T.S., Cooper K.D., Consolo M., Schastnaya J., Ozerov I.V., Aliper A. (2017). An analysis of gene expression data involving examination of signaling pathways activation reveals new insights into the mechanism of action of minoxidil topical foam in men with androgenetic alopecia. Cell Cycle.

[3] Shimizu Y., Ntege E.H., Sunami H., Inoue Y. (2022). Regenerative medicine strategies for hair growth and regeneration: A narrative review of literature. Regen. Ther..

[4] Oiwoh S.O., Enitan A.O., Adegbosin O.T., Akinboro A.O., Onayemi E.O. (2024). Androgenetic Alopecia: A Review. Niger. Postgrad. Med. J..

[5] Rosenthal A., Conde G., Greco J.F., Gharavi N.M. (2024). Management of androgenic alopecia: A systematic review of the literature. J. Cosmet. Laser Ther..

[6] Sekhavat H., Bar Yehuda S., Asotra S. (2025). Using the Mechanisms of Action Involved in the Pathogenesis of Androgenetic Alopecia to Treat Hair Loss. Int. J. Mol. Sci..

[7] Jain R., De-Eknamkul W. (2014). Potential targets in the discovery of new hair growth promoters for androgenic alopecia. Expert Opin. Ther. Targets.

[8] Jaworsky C., Kligman A.M., Murphy G.F. (1992). Characterization of inflammatory infiltrates in male pattern alopecia: Implications for pathogenesis. Br. J. Dermatol..

[9] Whiting D.A. (1993). Diagnostic and predictive value of horizontal sections of scalp biopsy specimens in male pattern androgenetic alopecia. J. Am. Acad. Dermatol..

[10] Mahé Y.F., Michelet J.F., Billoni N., Jarrousse F., Buan B., Commo S., Saint-Léger D., Bernard B.A. (2000). Androgenetic alopecia and microinflammation. Int. J. Dermatol..

[11] Chen S., Li L., Ding W., Zhu Y., Zhou N. (2025). Androgenetic Alopecia: An Update on Pathogenesis and Pharmacological Treatment. Drug Des. Dev. Ther..

[12] Xiao Y., Zhang Y., Deng S., Yang X., Yao X. (2025). Immune and Non-immune Interactions in the Pathogenesis of Androgenetic Alopecia. Clin. Rev. Allergy Immunol..

[13] Chew E.G.Y., Lim T.C., Leong M.F., Liu X., Sia Y.Y., Leong S.T., Yan-Jiang B.C., Stoecklin C., Borhan R., Heilmann-Heimbach S. (2022). Observations that suggest a contribution of altered dermal papilla mitochondrial function to androgenetic alopecia. Exp. Dermatol..

[14] Chen Q., Sun T., Wang J., Jia J., Yi Y.H., Chen Y.X., Miao Y., Hu Z.Q. (2019). Hydroxytyrosol prevents dermal papilla cells inflammation under oxidative stress by inducing autophagy. J. Biochem. Mol. Toxicol..

[15] Fortes C., Mastroeni S., Mannooranparampil T., Abeni D., Panebianco A. (2018). Mediterranean diet: Fresh herbs and fresh vegetables decrease the risk of Androgenetic Alopecia in males. Arch. Dermatol. Res..

[16] Gokce N., Basgoz N., Kenanoglu S., Akalin H., Ozkul Y., Ergoren M., Beccari T., Bertelli M., Dundar M. (2022). An overview of the genetic aspects of hair loss and its connection with nutrition. Prev. Med. Hyg. J..

[17] Martínez-Zamora L., Peñalver R., Ros G., Nieto G. (2021). Olive Tree Derivatives and Hydroxytyrosol: Their Potential Effects on Human Health and Its Use as Functional Ingredient in Meat. Foods.

[18] Bertelli M., Kiani A.K., Paolacci S., Manara E., Kurti D., Dhuli K., Bushati V., Miertus J., Pangallo D., Baglivo M. (2020). Hydroxytyrosol: A natural compound with promising pharmacological activities. J. Biotechnol..

[19] Serra G., Deiana M., Spencer J.P.E., Corona G. (2017). Olive Oil Phenolics Prevent Oxysterol-Induced Proinflammatory Cytokine Secretion and Reactive Oxygen Species Production in Human Peripheral Blood Mononuclear Cells, Through Modulation of p38 and JNK Pathways. Mol. Nutr. Food Res..

[20] Romana-Souza B., Saguie B.O., Pereira De Almeida Nogueira N., Paes M., Dos Santos Valença S., Atella G.C., Monte-Alto-Costa A. (2020). Oleic acid and hydroxytyrosol present in olive oil promote ROS and inflammatory response in normal cultures of murine dermal fibroblasts through the NF-κB and NRF2 pathways. Food Res. Int..

[21] Lolli F., Pallotti F., Rossi A., Fortuna M.C., Caro G., Lenzi A., Sansone A., Lombardo F. (2017). Androgenetic alopecia: A review. Endocrine.

[22] Saleem S. (2024). Targeting MAPK signaling: A promising approach for treating inflammatory lung disease. Pathol.—Res. Pract..

[23] Morgan M.J., Liu Z.-G. (2011). Crosstalk of reactive oxygen species and NF-κB signaling. Cell Res..

[24] Huang Z., Li Y., Xie Y., Fu H., Weng Z., Yuan J., Wu L., Lin W., Cao Y., Ding B. (2025). Jiawei Erzhiwan Ameliorates Androgenetic Alopecia by Regulating the SIRT1/JNK/p38 MAPK Pathway. Drug Des. Dev. Ther..

[25] Zhang L., Wang X., Lu X., Ma Y., Xin X., Xu X., Wang S., Hou Y. (2020). Tetramethylpyrazine enhanced the therapeutic effects of human umbilical cord mesenchymal stem cells in experimental autoimmune encephalomyelitis mice through Nrf2/HO-1 signaling pathway. Stem Cell Res. Ther..

[26] Peng S., Zhang B., Yao J., Duan D., Fang J. (2015). Dual protection of hydroxytyrosol, an olive oil polyphenol, against oxidative damage in PC12 cells. Food Funct..

[27] Zheng A., Li H., Xu J., Cao K., Li H., Pu W., Yang Z., Peng Y., Long J., Liu J. (2015). Hydroxytyrosol improves mitochondrial function and reduces oxidative stress in the brain of db/db mice: Role of AMP-activated protein kinase activation. Br. J. Nutr..

[28] Zhou J., Lei Y., Zhang S., Liu Y., Yi D. (2025). Panaxadiol Attenuates Neuronal Oxidative Stress and Apoptosis in Cerebral Ischemia/Reperfusion Injury via Regulation of the JAK3/STAT3/HIF-1α Signaling Pathway. CNS Neurosci. Ther..

[29] Rossi A., Anzalone A., Fortuna M.C., Caro G., Garelli V., Pranteda G., Carlesimo M. (2016). Multi-therapies in androgenetic alopecia: Review and clinical experiences. Dermatol. Ther..

[30] Huang Y., Ding X., Shen Y., He J., Teng Y., Yang X., Yu Y., Xu D., Tao X., Fan Y. (2026). Bletilla striata polysaccharide (BSP) promotes hair growth and suppresses oxidative stress and senescence of dermal papilla cells by inhibiting prostaglandin-endoperoxide synthase 2 (PTGS2). Int. J. Biol. Macromol..

[31] Zhou Z., Dun L., Yang Q., Tao J., Yu P., Xu H., Zhao N., Zheng N., An H., Yi P. (2023). Tongqiao Huoxue decoction alleviates neurological impairment following ischemic stroke via the PTGS2/NF-kappa B axis. Brain Res..

[32] Li R., Xie J., Xu W., Zhang L., Lin H., Huang W. (2022). LPS-induced PTGS2 manipulates the inflammatory response through trophoblast invasion in preeclampsia via NF-κB pathway. Reprod. Biol..

[33] Kang J.-I., Kim S.-C., Kim M.-K., Boo H.-J., Kim E.-J., Im G.-J., Kim Y.H., Hyun J.-W., Kang J.-H., Koh Y.-S. (2015). Effects of dihydrotestosterone on rat dermal papilla cells in vitro. Eur. J. Pharmacol..

[34] Müller-Decker K., Leder C., Neumann M., Neufang G., Marks F., Fürstenberger G., Bayerl C., Schweizer J. (2003). Expression of Cyclooxygenase Isozymes During Morphogenesis and Cycling of Pelage Hair Follicles in Mouse Skin: Precocious Onset of the First Catagen Phase and Alopecia upon Cyclooxygenase-2 Overexpression. J. Investig. Dermatol..

[35] Doiphode S., Lokhande K.B., Ghosh P., Swamy K.V., Nagar S. (2023). Dual inhibition of cyclooxygenase-2 (COX-2) and 5-lipoxygenase (5-LOX) by resveratrol derivatives in cancer therapy: In silico approach. J. Biomol. Struct. Dyn..

[36] Doiphode S., Ghosh P., Swamy K.V., Nagar S. (2026). An In Silico Study of Resveratrol Derivatives as Dual Inhibitors of Cyclooxygenase-2 (COX-2) and 5-Lipoxygenase (5-LOX) in Cancer Therapy. Biotechnol. Appl. Biochem..

[37] Xue C., Kang J., Zhu Q., Gao X., Wu X., Yu Y. (2025). Unraveling the mechanism of curcumin in coronary slow flow phenomenon through network pharmacology and molecular docking. Sci. Rep..

[38] Singh A., Soni U., Varadwaj P.K., Misra K., Rizvi S.I. (2025). Anti-inflammatory effect of curcumin in an accelerated senescence model of Wistar rat: An in vivo and in-silico study. J. Biomol. Struct. Dyn..

[39] Moetlediwa M.T., Ramashia R., Mangale M.B., Pheiffer C., Jack B.U., Salifu E.Y., Ramharack P. (2026). Identification of Enhanced Cyclooxygenase-2 (COX-2) Inhibitors Beyond Curcumin Through Virtual Screening to Target Inflammation-Related Metabolic Complications. Int. J. Mol. Sci..

[40] Clough E., Barrett T., E Wilhite S., Ledoux P., Evangelista C., Kim I.F., Tomashevsky M., A Marshall K., Phillippy K.H., Sherman P.M. (2024). NCBI GEO: Archive for gene expression and epigenomics data sets: 23-year update. Nucleic Acids Res..

[41] Love M.I., Huber W., Anders S. (2014). Moderated estimation of fold change and dispersion for RNA-seq data with DESeq2. Genome Biol..

[42] Liu Q., Tang Y., Huang Y., Wang J.A., Yang K., Zhang Y., Pu W., Liu J., Shi X., Ma Y. (2022). Insights into male androgenetic alopecia using comparative transcriptome profiling: Hypoxia-inducible factor-1 and Wnt/β-catenin signalling pathways. Br. J. Dermatol..

[43] Daina A., Michielin O., Zoete V. (2019). SwissTargetPrediction: Updated data and new features for efficient prediction of protein targets of small molecules. Nucleic Acids Res..

[44] Wang X., Shen Y., Wang S., Li S., Zhang W., Liu X., Lai L., Pei J., Li H. (2017). PharmMapper 2017 update: A web server for potential drug target identification with a comprehensive target pharmacophore database. Nucleic Acids Res..

[45] Liu T., Hwang L., Burley S.K., I Nitsche C., Southan C., Walters W.P., Gilson M.K. (2025). BindingDB in 2024: A FAIR knowledgebase of protein-small molecule binding data. Nucleic Acids Res..

[46] Szklarczyk D., Santos A., von Mering C., Jensen L.J., Bork P., Kuhn M. (2016). STITCH 5: Augmenting protein–chemical interaction networks with tissue and affinity data. Nucleic Acids Res..

[47] Davis A.P., Wiegers T.C., Sciaky D., Barkalow F., Wyatt B., Wiegers J., McMorran R., Abrar S., Mattingly C.J. (2025). Linking chemical data from the Comparative Toxicogenomics Database with adverse outcome pathways from the AOP-Wiki: A mechanistic data-oriented approach to help inform environmental health. F1000Research.

[48] Cannon M., Stevenson J., Stahl K., Basu R., Coffman A., Kiwala S., McMichael J.F., Kuzma K., Morrissey D., Cotto K. (2024). DGIdb 5.0: Rebuilding the drug–gene interaction database for precision medicine and drug discovery platforms. Nucleic Acids Res..

[49] Ru J., Li P., Wang J., Zhou W., Li B., Huang C., Li P., Guo Z., Tao W., Yang Y. (2014). TCMSP: A database of systems pharmacology for drug discovery from herbal medicines. J. Cheminform..

[50] Stelzer G., Rosen N., Plaschkes I., Zimmerman S., Twik M., Fishilevich S., Stein T.I., Nudel R., Lieder I., Mazor Y. (2016). The GeneCards Suite: From Gene Data Mining to Disease Genome Sequence Analyses. Curr. Protoc. Bioinform..

[51] Hunter F.M.I., Ioannidis H., Bento A.P., Bosc N., Corbett S., Felix E., Magarinos M.P., Manners E., Smit I.A., De Veij M. (2025). Drug and Clinical Candidate Drug Data in ChEMBL. J. Med. Chem..

[52] Keiser M.J., Roth B.L., Armbruster B.N., Ernsberger P., Irwin J.J., Shoichet B.K. (2007). Relating protein pharmacology by ligand chemistry. Nat. Biotechnol..

[53] Yao Z.-J., Dong J., Che Y.-J., Zhu M.-F., Wen M., Wang N.-N., Wang S., Lu A.-P., Cao D.-S. (2016). TargetNet: A web service for predicting potential drug–target interaction profiling via multi-target SAR models. J. Comput.-Aided Mol. Des..

[54] Bardou P., Mariette J., Escudié F., Djemiel C., Klopp C. (2014). jvenn: An interactive Venn diagram viewer. BMC Bioinform..

[55] Wu T., Hu E., Xu S., Chen M., Guo P., Dai Z., Feng T., Zhou L., Tang W., Zhan L. (2021). clusterProfiler 4.0: A universal enrichment tool for interpreting omics data. Innovation.

[56] Chen L., Zhang Y.H., Wang S., Zhang Y., Huang T., Cai Y.D. (2017). Prediction and analysis of essential genes using the enrichments of gene ontology and KEGG pathways. PLoS ONE.

[57] Szklarczyk D., Kirsch R., Koutrouli M., Nastou K., Mehryary F., Hachilif R., Gable A.L., Fang T., Doncheva N.T., Pyysalo S. (2023). The STRING database in 2023: Protein–protein association networks and functional enrichment analyses for any sequenced genome of interest. Nucleic Acids Res..

[58] Kim S., Chen J., Cheng T., Gindulyte A., He J., He S., Li Q., A Shoemaker B., A Thiessen P., Yu B. (2025). PubChem 2025 update. Nucleic Acids Res..

[59] Ahmad S., Jose da Costa Gonzales L., Bowler-Barnett E.H., Rice D.L., Kim M., Wijerathne S., Luciani A., Kandasaamy S., Luo J., Watkins X. (2025). The UniProt website API: Facilitating programmatic access to protein knowledge. Nucleic Acids Res..

[60] Burley S.K., Bhatt R., Bhikadiya C., Bi C., Biester A., Biswas P., Bittrich S., Blaumann S., Brown R., Chao H. (2025). Updated resources for exploring experimentally-determined PDB structures and Computed Structure Models at the RCSB Protein Data Bank. Nucleic Acids Res..

[61] Lemkul J.A. (2018). From Proteins to Perturbed Hamiltonians: A Suite of Tutorials for the GROMACS-2018 Molecular Simulation Package [Article v1.0]. Living J. Comput. Mol. Sci..

