# Supplementary material for: Exploring the Potential Role of Hydroxytyrosol in Androgenetic Alopecia: An Integrated Bioinformatics and Molecular Simulation Study
Source: Int J Mol Sci. 2026 May 28;27(11):4858. doi: 10.3390/ijms27114858 (PMC13256409; doi:10.3390/ijms27114858)
Supplement: Supplementary file 1 [file ijms-27-04858-s001.zip › ijms-4334068-supplementary.pdf]

Table S1. The list of differentially expressed mRNAs.

GeneName	padj	pvalue	log2FC	baseMean	lfcSE	stat	regulation
**CA2**	1.85E-14	9.31E-19	-0.79	837.25	0.09	-8.84	down
**SRGN**	2.60E-12	2.61E-16	1.33	326.13	0.16	8.19	up
**LCE1A**	1.34E-10	2.58E-14	1.29	372.80	0.17	7.62	up
**THY1**	1.34E-10	2.70E-14	0.70	348.68	0.09	7.61	up
**C3**	1.36E-10	3.41E-14	0.92	2015.74	0.12	7.58	up
**ALDH1A3**	1.43E-10	4.32E-14	0.76	461.40	0.10	7.55	up
**MAP2K6**	4.53E-10	1.59E-13	-0.55	379.35	0.08	-7.38	down
**LCE1B**	5.13E-10	2.06E-13	1.10	618.10	0.15	7.34	up
**TIMP1**	2.93E-09	1.32E-12	0.59	235.00	0.08	7.09	up
**PFKP**	4.60E-09	2.31E-12	0.73	1091.50	0.10	7.01	up
**NNMT**	5.66E-09	3.13E-12	1.24	468.90	0.18	6.97	up
**OSMR**	6.46E-09	3.90E-12	0.61	1799.86	0.09	6.94	up
**LCE1C**	7.05E-09	4.96E-12	1.07	860.91	0.16	6.91	up
**SOX9**	7.05E-09	4.83E-12	-0.50	6248.75	0.07	-6.91	down
**MET**	7.90E-09	5.96E-12	0.59	914.35	0.09	6.88	up
**VEGFA**	2.60E-08	2.22E-11	0.73	2880.15	0.11	6.69	up
**LCE1E**	2.67E-08	2.41E-11	1.29	181.80	0.19	6.68	up
**BHLHE22**	2.71E-08	2.58E-11	-1.22	148.30	0.18	-6.67	down
**RASD1**	2.87E-08	2.88E-11	-0.66	706.69	0.10	-6.65	down
**ERO1A**	3.52E-08	3.90E-11	0.61	2542.63	0.09	6.61	up
**GFPT2**	3.52E-08	3.78E-11	1.02	174.77	0.15	6.61	up
**SFRP2**	5.64E-08	6.65E-11	1.15	252.21	0.18	6.53	up
**ARG1**	6.16E-08	7.74E-11	0.75	997.63	0.12	6.51	up
**FREM2**	6.47E-08	8.46E-11	-0.63	1085.60	0.10	-6.49	down
**HOXC10**	8.12E-08	1.14E-10	-1.19	85.40	0.18	-6.45	down
**NPNT**	1.26E-07	1.96E-10	-0.86	700.56	0.13	-6.36	down
**MEDAG**	1.33E-07	2.13E-10	1.69	124.88	0.27	6.35	up
**ADCYAP1**	1.33E-07	2.21E-10	1.67	89.76	0.26	6.35	up
**RNF223**	1.41E-07	2.42E-10	0.79	157.30	0.12	6.33	up
**HMOX1**	1.42E-07	2.49E-10	0.72	1060.57	0.11	6.33	up
**PTPRZ1**	1.45E-07	2.70E-10	-0.55	1883.54	0.09	-6.32	down
**SOX18**	1.45E-07	2.65E-10	-1.11	84.39	0.18	-6.32	down
**SOSTDC1**	2.23E-07	4.38E-10	-0.90	1211.24	0.15	-6.24	down
**HOXC11**	3.14E-07	6.30E-10	-1.09	54.21	0.18	-6.18	down
**DACH1**	3.37E-07	6.94E-10	-0.83	1411.92	0.13	-6.17	down
**PPFIA4**	3.37E-07	7.12E-10	1.05	551.26	0.17	6.16	up
**DENND3**	3.44E-07	7.51E-10	0.70	817.22	0.11	6.15	up
**PTX3**	3.44E-07	7.60E-10	2.68	22.10	0.44	6.15	up
**SAMD4A**	4.03E-07	9.11E-10	0.87	1132.29	0.14	6.12	up
**ECM1**	5.24E-07	1.24E-09	0.71	561.10	0.12	6.08	up
**TMEM144**	5.24E-07	1.23E-09	-0.57	383.61	0.09	-6.08	down
**MT1M**	5.66E-07	1.36E-09	2.06	26.78	0.34	6.06	up

**PTGDS**	6.53E-07	1.61E-09	0.87	679.99	0.14	6.03	up
**IL6R**	6.61E-07	1.66E-09	0.60	772.67	0.10	6.03	up
**PCDH11X**	7.64E-07	1.96E-09	-0.78	214.82	0.13	-6.00	down
**SDCBP2**	8.61E-07	2.34E-09	0.61	997.69	0.10	5.97	up
**ARG2**	1.11E-06	3.07E-09	0.63	122.94	0.11	5.93	up
**NAMPTP1**	1.11E-06	3.12E-09	0.72	101.97	0.12	5.93	up
**FAM107A**	1.20E-06	3.43E-09	0.63	309.66	0.11	5.91	up
**C1R**	1.38E-06	4.10E-09	0.87	352.61	0.15	5.88	up
**AOX1**	1.42E-06	4.43E-09	0.79	270.20	0.13	5.87	up
**FLG**	1.53E-06	4.84E-09	0.65	121454.25	0.11	5.85	up
**EPOP**	1.65E-06	5.32E-09	1.19	138.05	0.20	5.84	up
**CTNND2**	2.00E-06	6.54E-09	-0.75	987.81	0.13	-5.80	down
**PRSS8**	2.63E-06	9.00E-09	0.50	2311.02	0.09	5.75	up
**BACH2**	2.75E-06	9.64E-09	0.80	945.53	0.14	5.74	up
**DEPTOR**	2.75E-06	9.67E-09	-0.61	360.09	0.11	-5.74	down
**TP73**	2.83E-06	1.01E-08	-0.73	518.28	0.13	-5.73	down
**TGFB2**	3.20E-06	1.16E-08	-0.99	632.21	0.17	-5.71	down
**SLC26A7**	3.35E-06	1.23E-08	-0.69	127.96	0.12	-5.70	down
**IL1RL1**	3.45E-06	1.30E-08	0.92	270.92	0.16	5.69	up
**ARHGAP28**	4.04E-06	1.54E-08	-0.54	1950.69	0.10	-5.66	down
**LCE5A**	4.21E-06	1.63E-08	1.40	89.52	0.25	5.65	up
**LHX2**	4.29E-06	1.70E-08	-0.85	735.16	0.15	-5.64	down
**LCE2D**	4.62E-06	1.86E-08	1.40	197.33	0.25	5.62	up
**VSNL1**	5.50E-06	2.27E-08	-0.51	1182.34	0.09	-5.59	down
**HRH1**	6.09E-06	2.54E-08	0.66	270.87	0.12	5.57	up
**CELF2**	6.13E-06	2.59E-08	0.54	749.19	0.10	5.57	up
**PPP1R16B**	6.29E-06	2.69E-08	0.60	159.19	0.11	5.56	up
**TRIL**	6.94E-06	3.14E-08	-0.51	853.41	0.09	-5.53	down
**TEDC1**	7.72E-06	3.53E-08	-0.91	281.57	0.16	-5.51	down
**IRAK3**	8.61E-06	4.16E-08	0.64	827.77	0.12	5.48	up
**CLDN8**	9.16E-06	4.51E-08	-0.86	279.97	0.16	-5.47	down
**PDE4A**	9.43E-06	4.81E-08	0.67	138.50	0.12	5.46	up
**TRPM6**	9.43E-06	4.81E-08	-0.63	1078.41	0.11	-5.46	down
**PCDHGA7**	1.01E-05	5.39E-08	-0.55	147.96	0.10	-5.44	down
**RNASE7**	1.25E-05	6.85E-08	1.07	411.60	0.20	5.40	up
**BIRC3**	1.29E-05	7.14E-08	0.93	818.54	0.17	5.39	up
**CYP1B1**	1.49E-05	8.31E-08	-0.73	2740.57	0.14	-5.36	down
**ELMOD1**	1.52E-05	8.69E-08	0.51	553.46	0.10	5.35	up
**NAMPT**	1.52E-05	8.62E-08	0.68	3654.94	0.13	5.35	up
**STK10**	1.52E-05	8.62E-08	0.52	1070.77	0.10	5.35	up
**SGIP1**	1.53E-05	8.84E-08	0.63	210.58	0.12	5.35	up
**KRT17P2**	1.88E-05	1.09E-07	-0.67	107.83	0.13	-5.31	down
**DPP10**	2.08E-05	1.23E-07	-0.68	134.82	0.13	-5.29	down
**SAMD9**	2.29E-05	1.38E-07	-0.69	784.73	0.13	-5.27	down

**SLC25A48**	2.37E-05	1.45E-07	-0.65	236.60	0.12	-5.26	down
**ABCC3**	2.66E-05	1.64E-07	0.79	1434.86	0.15	5.24	up
**PRRX2**	3.09E-05	1.96E-07	0.55	202.33	0.10	5.20	up
**ART3**	3.17E-05	2.07E-07	-0.69	103.19	0.13	-5.19	down
**ZDHHC11B**	3.20E-05	2.11E-07	0.59	274.32	0.11	5.19	up
**CA12**	3.47E-05	2.32E-07	0.53	3697.75	0.10	5.17	up
**ENO2**	3.51E-05	2.40E-07	0.54	246.18	0.11	5.17	up
**SYTL3**	3.64E-05	2.50E-07	0.80	176.05	0.16	5.16	up
**TRIB3**	3.70E-05	2.61E-07	0.72	156.38	0.14	5.15	up
**TMEM40**	3.71E-05	2.63E-07	0.53	1239.62	0.10	5.15	up
**HDC**	3.82E-05	2.75E-07	1.18	99.93	0.23	5.14	up
**NCCRP1**	3.88E-05	2.85E-07	0.64	2600.50	0.13	5.13	up
**AC254813.1**	4.10E-05	3.07E-07	0.65	136.21	0.13	5.12	up
**C1orf68**	4.42E-05	3.36E-07	0.92	508.58	0.18	5.10	up
**KCNH1**	4.44E-05	3.41E-07	-0.53	118.99	0.10	-5.10	down
**KPRP**	4.44E-05	3.48E-07	1.19	2249.51	0.23	5.10	up
**SLA**	4.45E-05	3.51E-07	0.83	322.46	0.16	5.09	up
**COCH**	4.53E-05	3.59E-07	-0.90	318.36	0.18	-5.09	down
**IL18R1**	4.75E-05	3.82E-07	0.76	230.73	0.15	5.08	up
**BARX2**	4.80E-05	3.89E-07	-0.54	2378.28	0.11	-5.07	down
**LMX1B**	4.98E-05	4.15E-07	-0.62	226.65	0.12	-5.06	down
**NDRG1**	4.98E-05	4.15E-07	0.62	12009.81	0.12	5.06	up
**TACC1**	4.98E-05	4.14E-07	0.53	2377.37	0.10	5.06	up
**PFKFB3**	5.13E-05	4.33E-07	0.64	2554.28	0.13	5.05	up
**CRCT1**	5.59E-05	4.83E-07	0.70	1105.91	0.14	5.03	up
**LAPTM5**	5.59E-05	4.83E-07	0.80	400.08	0.16	5.03	up
**ATP10A**	5.75E-05	5.00E-07	0.59	297.65	0.12	5.03	up
**ST3GAL1**	6.14E-05	5.37E-07	0.52	670.01	0.10	5.01	up
**LCE1D**	6.15E-05	5.41E-07	1.14	93.75	0.23	5.01	up
**SHISA2**	6.18E-05	5.46E-07	-0.88	850.34	0.18	-5.01	down
**MT1A**	6.30E-05	5.61E-07	3.15	25.51	0.63	5.00	up
**CP**	6.39E-05	5.75E-07	0.67	154.71	0.13	5.00	up
**ASPHD2**	6.43E-05	5.93E-07	0.75	156.92	0.15	4.99	up
**LCE2A**	6.43E-05	5.90E-07	1.27	193.82	0.25	4.99	up
**PDZRN3**	6.43E-05	5.93E-07	-0.71	5709.11	0.14	-4.99	down
**AC129492.6**	7.22E-05	6.75E-07	1.06	199.51	0.21	4.97	up
**MAMDC2**	7.26E-05	6.82E-07	-0.66	544.56	0.13	-4.97	down
**C7**	7.33E-05	6.93E-07	1.05	127.67	0.21	4.96	up
**GPC5**	7.36E-05	7.01E-07	-0.73	58.99	0.15	-4.96	down
**LCE2B**	7.36E-05	7.03E-07	1.32	506.78	0.27	4.96	up
**CD53**	7.55E-05	7.24E-07	0.85	141.94	0.17	4.95	up
**HOXB3**	7.95E-05	7.67E-07	-0.80	66.09	0.16	-4.94	down
**PTPRT**	8.07E-05	7.83E-07	-0.51	341.01	0.10	-4.94	down
**GRIA4**	8.28E-05	8.15E-07	-0.72	136.79	0.14	-4.93	down

**PHYHIPL**	8.43E-05	8.35E-07	-0.78	97.81	0.16	-4.93	down
**RUBCNL**	8.66E-05	8.71E-07	0.84	78.95	0.17	4.92	up
**PPP1R1C**	8.74E-05	8.83E-07	-0.95	166.77	0.19	-4.92	down
**HSPA2**	8.94E-05	9.07E-07	-0.70	2565.50	0.14	-4.91	down
**ADIPOQ**	9.62E-05	9.91E-07	-0.84	115.91	0.17	-4.89	down
**CDH12**	9.89E-05	1.04E-06	-0.79	127.63	0.16	-4.88	down
**IL37**	9.89E-05	1.04E-06	0.73	154.89	0.15	4.88	up
**RPL9P16**	1.01E-04	1.07E-06	-0.70	119.64	0.14	-4.88	down
**MMP19**	1.01E-04	1.08E-06	0.65	241.94	0.13	4.88	up
**TSSK1B**	1.01E-04	1.08E-06	-0.83	76.39	0.17	-4.88	down
**ALOX5**	1.05E-04	1.13E-06	0.59	167.45	0.12	4.87	up
**SEZ6L**	1.06E-04	1.15E-06	-0.63	252.75	0.13	-4.86	down
**IGLV3-21**	1.08E-04	1.17E-06	2.78	11.75	0.57	4.86	up
**NPPC**	1.11E-04	1.22E-06	-1.39	52.11	0.29	-4.85	down
**TBX18**	1.21E-04	1.37E-06	-0.74	310.16	0.15	-4.83	down
**PFKFB4**	1.28E-04	1.45E-06	0.71	685.07	0.15	4.82	up
**PRKCB**	1.29E-04	1.49E-06	0.94	184.77	0.20	4.81	up
**EFNA5**	1.29E-04	1.52E-06	-0.63	2568.60	0.13	-4.81	down
**AL033519.2**	1.30E-04	1.54E-06	1.27	33.44	0.27	4.81	up
**TBC1D2**	1.31E-04	1.56E-06	0.52	502.06	0.11	4.80	up
**STXBP6**	1.32E-04	1.58E-06	0.70	242.44	0.15	4.80	up
**ATP8A2**	1.35E-04	1.64E-06	-0.80	352.31	0.17	-4.79	down
**SYTL5**	1.43E-04	1.75E-06	-0.78	139.96	0.16	-4.78	down
**TPSAB1**	1.44E-04	1.77E-06	0.79	169.47	0.17	4.78	up
**EGLN3**	1.46E-04	1.80E-06	0.57	2053.98	0.12	4.77	up
**GAS1**	1.57E-04	2.01E-06	-0.63	1280.41	0.13	-4.75	down
**FUT3**	1.58E-04	2.03E-06	0.71	162.09	0.15	4.75	up
**AZGP1P1**	1.80E-04	2.37E-06	0.64	265.15	0.14	4.72	up
**GASK1B**	1.88E-04	2.49E-06	-0.55	1124.77	0.12	-4.71	down
**CA9**	2.30E-04	3.14E-06	0.94	88.86	0.20	4.66	up
**OXGR1**	2.30E-04	3.16E-06	-0.93	113.90	0.20	-4.66	down
**LCE2C**	2.38E-04	3.29E-06	1.40	375.52	0.30	4.65	up
**ZFP36L1**	2.40E-04	3.32E-06	-0.53	8596.55	0.11	-4.65	down
**LCE6A**	2.40E-04	3.34E-06	0.56	635.67	0.12	4.65	up
**IKZF1**	2.66E-04	3.81E-06	0.96	145.37	0.21	4.62	up
**RBBP8NL**	2.66E-04	3.83E-06	-0.72	163.64	0.16	-4.62	down
**COL17A1**	2.75E-04	4.00E-06	0.52	1462.46	0.11	4.61	up
**C1QTNF3**	2.82E-04	4.14E-06	-0.50	156.16	0.11	-4.60	down
**RUNX1T1**	2.82E-04	4.15E-06	-0.53	319.28	0.11	-4.60	down
**CBLN2**	3.15E-04	4.66E-06	-1.14	927.11	0.25	-4.58	down
**PARM1**	3.32E-04	4.94E-06	-0.61	4080.73	0.13	-4.57	down
**CRISPLD2**	3.43E-04	5.14E-06	0.61	1508.14	0.13	4.56	up
**HBB**	3.43E-04	5.18E-06	2.31	397.39	0.51	4.56	up
**TNS4**	3.43E-04	5.15E-06	0.54	7235.50	0.12	4.56	up

**PLIN1**	3.58E-04	5.47E-06	-0.56	107.89	0.12	-4.55	down
**NPY1R**	3.81E-04	5.98E-06	-0.65	251.79	0.14	-4.53	down
**LOX**	3.85E-04	6.10E-06	0.66	487.84	0.15	4.52	up
**LCE1F**	3.94E-04	6.28E-06	1.06	100.27	0.23	4.52	up
**ARPP21**	4.01E-04	6.41E-06	-1.32	53.72	0.29	-4.51	down
**HOXC9**	4.04E-04	6.50E-06	-1.15	60.91	0.25	-4.51	down
**HSPA12B**	4.22E-04	6.92E-06	-0.60	93.56	0.13	-4.50	down
**ATF3**	4.30E-04	7.06E-06	-1.72	876.12	0.38	-4.49	down
**HBA2**	4.32E-04	7.20E-06	2.34	396.18	0.52	4.49	up
**PIK3R5**	4.32E-04	7.21E-06	0.77	101.27	0.17	4.49	up
**BTBD8**	4.44E-04	7.43E-06	-0.60	152.92	0.13	-4.48	down
**AKAP12**	4.56E-04	7.67E-06	0.60	486.06	0.13	4.47	up
**BTC**	4.71E-04	8.01E-06	-0.67	598.10	0.15	-4.47	down
**SLC26A5**	4.78E-04	8.17E-06	-0.72	121.66	0.16	-4.46	down
**APLNR**	4.90E-04	8.42E-06	-0.95	113.39	0.21	-4.45	down
**FCGR2A**	4.94E-04	8.56E-06	0.59	260.18	0.13	4.45	up
**STMN2**	4.94E-04	8.62E-06	1.04	46.07	0.23	4.45	up
**ERRFI1**	5.01E-04	8.78E-06	0.70	1481.01	0.16	4.45	up
**KLF9**	5.07E-04	8.95E-06	0.75	960.62	0.17	4.44	up
**MT1E**	5.26E-04	9.35E-06	0.91	289.04	0.21	4.43	up
**CCR7**	5.26E-04	9.38E-06	1.11	42.48	0.25	4.43	up
**CD163**	5.42E-04	9.81E-06	0.71	345.56	0.16	4.42	up
**KCNH5**	5.49E-04	1.00E-05	-0.54	96.62	0.12	-4.42	down
**KCNH8**	5.71E-04	1.05E-05	-0.57	166.69	0.13	-4.41	down
**PDK4**	5.73E-04	1.07E-05	0.74	1345.20	0.17	4.40	up
**RIMS2**	5.73E-04	1.06E-05	-0.59	735.89	0.13	-4.40	down
**ODF3L1**	6.05E-04	1.16E-05	-1.16	25.17	0.27	-4.38	down
**PTGS2**	6.05E-04	1.15E-05	-0.92	283.09	0.21	-4.39	down
**RFLNA**	6.05E-04	1.16E-05	-0.87	37.45	0.20	-4.39	down
**ICAM1**	6.16E-04	1.19E-05	0.50	231.99	0.11	4.38	up
**SLC35F3**	6.19E-04	1.20E-05	-0.65	139.57	0.15	-4.38	down
**MT1X**	6.24E-04	1.22E-05	1.06	2105.38	0.24	4.37	up
**TCAF2P1**	6.24E-04	1.22E-05	0.91	34.43	0.21	4.37	up
**CHST2**	7.16E-04	1.43E-05	-0.67	2178.93	0.16	-4.34	down
**HOXC4**	7.57E-04	1.54E-05	-0.86	64.05	0.20	-4.32	down
**HILPDA**	7.70E-04	1.57E-05	0.65	896.51	0.15	4.32	up
**ADPRHL1**	7.76E-04	1.58E-05	0.85	127.34	0.20	4.32	up
**ADM**	7.90E-04	1.62E-05	0.67	443.79	0.16	4.31	up
**UNC5A**	8.53E-04	1.77E-05	-1.06	88.67	0.25	-4.29	down
**SAMSN1**	8.56E-04	1.78E-05	0.86	76.41	0.20	4.29	up
**NMNAT1P1**	8.64E-04	1.80E-05	-1.42	15.59	0.33	-4.29	down
**HPGD**	9.01E-04	1.89E-05	0.65	822.12	0.15	4.28	up
**FOS**	9.09E-04	1.92E-05	-2.17	5195.93	0.51	-4.27	down
**IGHA1**	9.13E-04	1.94E-05	1.54	75.67	0.36	4.27	up

**MEOX2**	9.68E-04	2.08E-05	-0.87	101.22	0.20	-4.26	down
**CCN4**	9.98E-04	2.15E-05	0.70	122.59	0.16	4.25	up
**AL928654.3**	1.02E-03	2.22E-05	-1.25	45.33	0.30	-4.24	down
**ANKRD33B**	1.09E-03	2.40E-05	0.51	1045.04	0.12	4.22	up
**ZNF727**	1.14E-03	2.55E-05	-0.83	51.21	0.20	-4.21	down
**NRXN1**	1.22E-03	2.76E-05	-0.55	289.27	0.13	-4.19	down
**VTCN1**	1.22E-03	2.77E-05	-0.56	449.44	0.13	-4.19	down
**TNFSF8**	1.24E-03	2.82E-05	1.05	28.58	0.25	4.19	up
**DDIT3**	1.27E-03	2.93E-05	0.57	153.44	0.14	4.18	up
**TBX2**	1.27E-03	2.93E-05	-0.61	111.63	0.14	-4.18	down
**FAM153B**	1.33E-03	3.10E-05	0.69	102.19	0.16	4.17	up
**COL6A4P2**	1.34E-03	3.14E-05	-0.73	118.60	0.17	-4.16	down
**S100A1**	1.41E-03	3.33E-05	-0.58	275.65	0.14	-4.15	down
**AC027644.4**	1.43E-03	3.41E-05	0.68	60.21	0.16	4.14	up
**HOXC6**	1.43E-03	3.42E-05	-0.83	110.16	0.20	-4.14	down
**COL5A3**	1.44E-03	3.45E-05	-0.57	1439.51	0.14	-4.14	down
**WFDC5**	1.46E-03	3.53E-05	0.56	574.01	0.14	4.14	up
**HCLS1**	1.50E-03	3.67E-05	0.70	212.67	0.17	4.13	up
**RPL21P109**	1.50E-03	3.70E-05	-1.30	26.99	0.31	-4.13	down
**GYPC**	1.52E-03	3.78E-05	0.64	106.58	0.16	4.12	up
**NDNF**	1.54E-03	3.90E-05	-0.57	62.20	0.14	-4.11	down
**DNAH17**	1.55E-03	3.94E-05	0.59	2384.30	0.14	4.11	up
**ARHGAP9**	1.57E-03	4.03E-05	0.58	87.32	0.14	4.11	up
**FOSB**	1.59E-03	4.13E-05	-2.37	3650.39	0.58	-4.10	down
**CPA3**	1.60E-03	4.18E-05	0.64	204.48	0.16	4.10	up
**FOLH1**	1.62E-03	4.23E-05	-0.63	125.28	0.15	-4.09	down
**SMIM3**	1.66E-03	4.38E-05	0.96	56.61	0.23	4.09	up
**FAM89A**	1.69E-03	4.48E-05	0.62	215.47	0.15	4.08	up
**GPAT3**	1.77E-03	4.72E-05	0.59	100.00	0.15	4.07	up
**TUBB1**	1.80E-03	4.86E-05	0.61	45.83	0.15	4.06	up
**CRHR1**	1.85E-03	5.04E-05	-0.93	46.82	0.23	-4.05	down
**SDC4P**	1.89E-03	5.18E-05	1.25	21.64	0.31	4.05	up
**HAL**	1.90E-03	5.22E-05	0.85	1386.46	0.21	4.05	up
**CCR1**	1.90E-03	5.23E-05	0.80	32.04	0.20	4.05	up
**IL18RAP**	2.02E-03	5.59E-05	1.57	13.03	0.39	4.03	up
**AL441963.1**	2.07E-03	5.79E-05	-0.58	59.13	0.14	-4.02	down
**IGKV1D-39**	2.10E-03	5.93E-05	1.75	22.63	0.44	4.02	up
**ALKAL2**	2.14E-03	6.03E-05	0.88	50.36	0.22	4.01	up
**IFI30**	2.27E-03	6.47E-05	0.56	254.97	0.14	4.00	up
**IQCN**	2.27E-03	6.49E-05	-0.52	491.97	0.13	-3.99	down
**ATP5MC1P6**	2.28E-03	6.52E-05	-0.69	95.20	0.17	-3.99	down
**PAX1**	2.28E-03	6.52E-05	0.79	55.10	0.20	3.99	up
**USP2**	2.28E-03	6.55E-05	0.54	1031.24	0.14	3.99	up
**ATRNL1**	2.34E-03	6.77E-05	0.71	199.92	0.18	3.98	up

**LOR**	2.43E-03	7.05E-05	0.53	1454.07	0.13	3.97	up
**AL136982.5**	2.44E-03	7.11E-05	0.76	45.87	0.19	3.97	up
**HOXC5**	2.44E-03	7.10E-05	-1.14	31.71	0.29	-3.97	down
**GADD45B**	2.51E-03	7.41E-05	0.64	479.67	0.16	3.96	up
**NR2F2**	2.52E-03	7.46E-05	-0.51	314.08	0.13	-3.96	down
**BNC2**	2.65E-03	7.90E-05	-0.61	2993.63	0.15	-3.95	down
**ADAMTS9**	2.69E-03	8.02E-05	0.52	887.76	0.13	3.94	up
**DUSP1**	2.73E-03	8.20E-05	-1.26	5144.64	0.32	-3.94	down
**TMEM132A**	2.74E-03	8.27E-05	-0.56	809.08	0.14	-3.94	down
**LGR5**	2.78E-03	8.42E-05	-0.84	1840.49	0.21	-3.93	down
**IL7R**	2.92E-03	8.98E-05	1.04	455.40	0.27	3.92	up
**HSPA6**	2.93E-03	9.05E-05	0.63	73.01	0.16	3.91	up
**RIPOR3**	3.00E-03	9.30E-05	0.51	386.54	0.13	3.91	up
**IGHG4**	3.07E-03	9.57E-05	1.94	38.04	0.50	3.90	up
**TUBB2BP1**	3.15E-03	9.89E-05	-1.50	26.47	0.38	-3.89	down
**ZBTB16**	3.19E-03	1.00E-04	0.89	1704.41	0.23	3.89	up
**TRPM5**	3.19E-03	1.01E-04	-0.84	72.37	0.21	-3.89	down
**AC011473.4**	3.21E-03	1.02E-04	1.31	262.38	0.34	3.89	up
**SLITRK5**	3.22E-03	1.02E-04	-0.59	208.11	0.15	-3.89	down
**SEMA3A**	3.22E-03	1.02E-04	0.61	98.63	0.16	3.88	up
**HBA1**	3.26E-03	1.04E-04	2.14	301.42	0.55	3.88	up
**LCP2**	3.26E-03	1.04E-04	0.80	154.40	0.21	3.88	up
**SOX17**	3.28E-03	1.05E-04	-0.81	40.25	0.21	-3.88	down
**BATF**	3.41E-03	1.11E-04	1.57	21.67	0.41	3.87	up
**COL9A2**	3.41E-03	1.11E-04	-0.57	462.90	0.15	-3.87	down
**TNFRSF6B**	3.45E-03	1.12E-04	1.43	33.75	0.37	3.86	up
**RIBC2**	3.51E-03	1.16E-04	-0.70	32.34	0.18	-3.86	down
**FRG2HP**	3.66E-03	1.21E-04	-0.58	404.58	0.15	-3.84	down
**CSRNP3**	3.67E-03	1.22E-04	-0.52	376.80	0.14	-3.84	down
**SLAMF1**	3.71E-03	1.24E-04	1.31	41.65	0.34	3.84	up
**SLC39A14**	3.89E-03	1.31E-04	0.57	998.86	0.15	3.82	up
**BTLA**	3.96E-03	1.35E-04	0.67	40.80	0.17	3.82	up
**CTSH**	4.04E-03	1.38E-04	0.52	1407.89	0.14	3.81	up
**SCN2B**	4.07E-03	1.40E-04	-0.54	84.59	0.14	-3.81	down
**MRC2**	4.28E-03	1.51E-04	-0.54	5706.42	0.14	-3.79	down
**WFDC21P**	4.46E-03	1.58E-04	0.58	204.19	0.15	3.78	up
**PCSK1**	4.53E-03	1.62E-04	-0.52	262.23	0.14	-3.77	down
**ZKSCAN7**	4.58E-03	1.64E-04	-0.56	105.13	0.15	-3.77	down
**SH3RF3**	4.71E-03	1.70E-04	0.50	243.86	0.13	3.76	up
**SLC46A2**	4.71E-03	1.70E-04	0.54	233.00	0.14	3.76	up
**SHQ1P1**	4.77E-03	1.73E-04	-0.56	82.13	0.15	-3.76	down
**SERPINB12**	4.80E-03	1.74E-04	0.60	667.07	0.16	3.75	up
**CST7**	4.80E-03	1.75E-04	0.71	60.11	0.19	3.75	up
**PSG7**	4.89E-03	1.79E-04	0.64	139.72	0.17	3.75	up

**FKBP5**	4.91E-03	1.80E-04	0.91	3992.41	0.24	3.75	up
**EGR2**	5.04E-03	1.87E-04	-0.53	1611.17	0.14	-3.74	down
**P3H2**	5.04E-03	1.88E-04	0.54	548.53	0.15	3.74	up
**CD164L2**	5.05E-03	1.88E-04	0.62	80.12	0.17	3.73	up
**ADRB2**	5.09E-03	1.91E-04	0.61	297.16	0.16	3.73	up
**GAS2**	5.09E-03	1.91E-04	-0.65	85.93	0.17	-3.73	down
**L1TD1**	5.17E-03	1.95E-04	-0.69	46.51	0.19	-3.73	down
**PGLYRP2**	5.17E-03	1.95E-04	1.01	16.52	0.27	3.73	up
**FGR**	5.22E-03	1.98E-04	0.54	117.34	0.14	3.72	up
**HINT2P1**	5.28E-03	2.01E-04	-1.09	19.41	0.29	-3.72	down
**IGKC**	5.80E-03	2.29E-04	1.47	370.70	0.40	3.68	up
**KCNJ18**	6.11E-03	2.45E-04	0.67	85.43	0.18	3.67	up
**YOD1**	6.12E-03	2.45E-04	0.53	2428.71	0.14	3.67	up
**SASH3**	6.15E-03	2.47E-04	0.75	63.21	0.21	3.67	up
**SLC41A1**	6.17E-03	2.48E-04	0.52	1184.11	0.14	3.66	up
**CYTIP**	6.29E-03	2.57E-04	0.70	133.20	0.19	3.66	up
**STC1**	6.30E-03	2.57E-04	0.84	177.81	0.23	3.65	up
**LCT**	6.35E-03	2.60E-04	0.99	20.31	0.27	3.65	up
**PHF2P2**	6.35E-03	2.61E-04	0.89	36.94	0.24	3.65	up
**OLFM4**	6.39E-03	2.62E-04	-1.55	24.71	0.43	-3.65	down
**ADAMTS15**	6.49E-03	2.67E-04	-0.50	2198.27	0.14	-3.65	down
**SIK1**	6.52E-03	2.70E-04	-0.98	701.73	0.27	-3.64	down
**DAB1**	6.62E-03	2.75E-04	-0.57	90.43	0.16	-3.64	down
**TMEM163**	6.93E-03	2.94E-04	-0.58	88.15	0.16	-3.62	down
**JUN**	6.94E-03	2.95E-04	-0.71	3433.62	0.20	-3.62	down
**IGHG3**	6.94E-03	2.96E-04	1.69	22.47	0.47	3.62	up
**CHRNA9**	7.01E-03	3.00E-04	0.63	127.86	0.17	3.62	up
**TENT5C**	7.02E-03	3.01E-04	0.50	392.32	0.14	3.61	up
**SLC7A4**	7.19E-03	3.10E-04	-0.63	161.24	0.17	-3.61	down
**IGKV4-1**	7.24E-03	3.14E-04	1.62	17.92	0.45	3.60	up
**WIF1**	7.42E-03	3.24E-04	-0.79	75.56	0.22	-3.59	down
**TGM4**	7.66E-03	3.39E-04	0.86	27.74	0.24	3.58	up
**SPOCK2**	7.70E-03	3.43E-04	0.69	167.61	0.19	3.58	up
**PSPC1P1**	7.72E-03	3.45E-04	-1.21	12.05	0.34	-3.58	down
**KITLG**	7.80E-03	3.49E-04	-0.52	512.32	0.14	-3.58	down
**SIX1**	8.12E-03	3.69E-04	1.68	11.12	0.47	3.56	up
**TMEM107**	8.12E-03	3.70E-04	-0.52	257.29	0.15	-3.56	down
**JAK3**	8.25E-03	3.77E-04	0.62	185.28	0.17	3.56	up
**CHRNA3**	8.28E-03	3.79E-04	0.50	68.80	0.14	3.55	up
**C2orf72**	8.45E-03	3.90E-04	-0.79	38.51	0.22	-3.55	down
**AL355922.2**	8.48E-03	3.93E-04	-0.68	36.76	0.19	-3.54	down
**CXCR4**	8.48E-03	3.93E-04	0.66	187.36	0.19	3.54	up
**SERPINE1**	8.60E-03	3.99E-04	0.67	329.37	0.19	3.54	up
**IGHG1**	8.93E-03	4.23E-04	1.63	357.12	0.46	3.53	up

**THEMIS2**	8.93E-03	4.23E-04	0.53	171.38	0.15	3.53	up
**TMC5**	8.95E-03	4.27E-04	0.55	340.92	0.16	3.52	up
**FAM218A**	9.00E-03	4.30E-04	-0.59	82.91	0.17	-3.52	down
**ROPN1**	9.28E-03	4.47E-04	-0.57	89.23	0.16	-3.51	down
**SLAMF8**	9.31E-03	4.50E-04	0.88	46.60	0.25	3.51	up
**RPSAP75**	9.35E-03	4.53E-04	-1.07	18.16	0.31	-3.51	down
**UCN2**	9.52E-03	4.65E-04	-0.50	117.09	0.14	-3.50	down
**AL049757.1**	9.52E-03	4.66E-04	0.85	17.66	0.24	3.50	up
**SLC16A9**	9.53E-03	4.67E-04	-0.58	256.75	0.17	-3.50	down
**LCE3D**	9.73E-03	4.81E-04	0.62	712.51	0.18	3.49	up
**AMN**	9.77E-03	4.83E-04	0.57	89.92	0.16	3.49	up
**AC012254.2**	9.93E-03	4.94E-04	2.15	18.81	0.62	3.48	up
**MPZ**	1.01E-02	5.04E-04	-0.55	67.19	0.16	-3.48	down
**KRT16P3**	1.03E-02	5.21E-04	-0.83	59.90	0.24	-3.47	down
**AC007376.1**	1.06E-02	5.42E-04	-0.89	16.63	0.26	-3.46	down
**EGR1**	1.09E-02	5.61E-04	-0.85	3568.68	0.25	-3.45	down
**COL9A3**	1.09E-02	5.66E-04	-0.66	114.82	0.19	-3.45	down
**IGLC1**	1.10E-02	5.67E-04	1.94	22.14	0.56	3.45	up
**RBMY2AP**	1.12E-02	5.82E-04	1.11	23.40	0.32	3.44	up
**C1QB**	1.17E-02	6.22E-04	0.59	85.75	0.17	3.42	up
**GRIA2**	1.21E-02	6.56E-04	-0.55	100.29	0.16	-3.41	down
**RBMY2CP**	1.23E-02	6.70E-04	1.00	24.69	0.30	3.40	up
**RBMY2DP**	1.23E-02	6.70E-04	1.00	24.69	0.30	3.40	up
**AC063952.1**	1.23E-02	6.72E-04	0.66	41.36	0.19	3.40	up
**SPRR5**	1.23E-02	6.71E-04	0.75	294.07	0.22	3.40	up
**PNLDC1**	1.24E-02	6.76E-04	0.62	487.37	0.18	3.40	up
**MOGAT1**	1.25E-02	6.89E-04	0.61	348.60	0.18	3.39	up
**TNFRSF12A**	1.26E-02	6.96E-04	0.59	374.19	0.17	3.39	up
**CTAGE6**	1.30E-02	7.23E-04	1.00	30.91	0.30	3.38	up
**PTCH2**	1.34E-02	7.58E-04	-0.60	624.61	0.18	-3.37	down
**MT2A**	1.36E-02	7.69E-04	1.12	1386.97	0.33	3.36	up
**SERPINA9**	1.38E-02	7.88E-04	0.61	181.05	0.18	3.36	up
**SYNPO2**	1.39E-02	7.92E-04	0.54	1701.17	0.16	3.36	up
**NLRP10**	1.42E-02	8.12E-04	0.54	175.27	0.16	3.35	up
**ABCG4**	1.45E-02	8.40E-04	0.90	49.51	0.27	3.34	up
**RASSF9**	1.46E-02	8.50E-04	-0.51	817.44	0.15	-3.34	down
**CD163L1**	1.47E-02	8.54E-04	0.63	84.49	0.19	3.33	up
**NRARP**	1.47E-02	8.59E-04	-0.53	581.83	0.16	-3.33	down
**RHCE**	1.49E-02	8.67E-04	0.69	25.81	0.21	3.33	up
**TSPAN8**	1.50E-02	8.88E-04	-0.58	120.75	0.17	-3.32	down
**GADD45A**	1.50E-02	8.90E-04	0.57	261.73	0.17	3.32	up
**ADGRB1**	1.52E-02	9.00E-04	-0.68	2719.64	0.21	-3.32	down
**PPP1R3C**	1.52E-02	9.01E-04	-0.63	403.76	0.19	-3.32	down
**MMP23B**	1.53E-02	9.10E-04	0.57	48.88	0.17	3.32	up

**KBTBD11**	1.58E-02	9.47E-04	0.54	99.64	0.16	3.31	up
**CCN5**	1.58E-02	9.50E-04	0.56	108.28	0.17	3.31	up
**RPSAP58**	1.63E-02	1.00E-03	0.52	113.31	0.16	3.29	up
**NAP1L2**	1.68E-02	1.04E-03	-0.74	57.67	0.23	-3.28	down
**KLF2**	1.73E-02	1.09E-03	-0.72	334.71	0.22	-3.27	down
**CNFN**	1.73E-02	1.09E-03	0.51	1260.06	0.16	3.27	up
**IGHV1-69D**	1.76E-02	1.12E-03	1.34	12.53	0.41	3.26	up
**SEC14L4**	1.76E-02	1.11E-03	0.52	388.42	0.16	3.26	up
**C1QC**	1.79E-02	1.14E-03	0.51	112.49	0.16	3.25	up
**HOXB4**	1.81E-02	1.17E-03	-0.88	21.46	0.27	-3.25	down
**CAMK4**	1.82E-02	1.17E-03	0.52	117.16	0.16	3.24	up
**IGHEP1**	1.82E-02	1.18E-03	1.42	15.52	0.44	3.24	up
**FMO6P**	1.83E-02	1.18E-03	-0.70	28.41	0.21	-3.24	down
**ELOVL5**	1.83E-02	1.18E-03	0.52	6439.46	0.16	3.24	up
**STON1-GTF2A1L**	1.84E-02	1.19E-03	-1.01	43.37	0.31	-3.24	down
**AGAP7P**	1.85E-02	1.20E-03	1.01	16.05	0.31	3.24	up
**RTL1**	1.87E-02	1.22E-03	1.02	43.27	0.32	3.23	up
**ELFN1**	1.89E-02	1.24E-03	-0.54	140.70	0.17	-3.23	down
**CCL19**	1.92E-02	1.27E-03	0.86	159.14	0.27	3.22	up
**MS4A1**	1.92E-02	1.28E-03	2.52	32.75	0.78	3.22	up
**SERPINF2**	1.92E-02	1.27E-03	0.68	86.21	0.21	3.22	up
**HCST**	1.94E-02	1.29E-03	1.01	16.62	0.31	3.22	up
**KLK8**	1.98E-02	1.33E-03	0.52	425.20	0.16	3.21	up
**DKK1**	2.01E-02	1.36E-03	1.26	22.56	0.39	3.20	up
**RHBDL1**	2.03E-02	1.40E-03	-0.51	49.26	0.16	-3.20	down
**PWWP3B**	2.07E-02	1.45E-03	-0.64	116.40	0.20	-3.19	down
**POMC**	2.08E-02	1.45E-03	-0.57	134.60	0.18	-3.18	down
**RPL35AP31**	2.09E-02	1.47E-03	-0.73	57.51	0.23	-3.18	down
**IGFBP3**	2.17E-02	1.55E-03	0.51	1441.27	0.16	3.17	up
**NOTCH1**	2.18E-02	1.56E-03	-0.54	417.45	0.17	-3.16	down
**ZNF177**	2.19E-02	1.57E-03	-0.83	54.30	0.26	-3.16	down
**IGLC2**	2.19E-02	1.57E-03	1.84	98.34	0.58	3.16	up
**MTCYBP44**	2.26E-02	1.63E-03	-0.55	84.33	0.17	-3.15	down
**AC025030.1**	2.27E-02	1.65E-03	-0.67	26.73	0.21	-3.15	down
**SLC22A13**	2.33E-02	1.71E-03	0.76	20.94	0.24	3.14	up
**CD7**	2.33E-02	1.71E-03	1.11	16.79	0.36	3.14	up
**MTATP6P11**	2.34E-02	1.73E-03	1.05	21.04	0.33	3.13	up
**CLDN17**	2.38E-02	1.77E-03	-1.11	23.72	0.35	-3.13	down
**C11orf42**	2.41E-02	1.80E-03	0.70	24.64	0.22	3.12	up
**AC010724.1**	2.43E-02	1.81E-03	0.99	13.19	0.32	3.12	up
**CCN2**	2.50E-02	1.89E-03	0.81	1728.26	0.26	3.11	up
**ASIP**	2.51E-02	1.90E-03	0.78	21.29	0.25	3.10	up
**PIRT**	2.53E-02	1.93E-03	-0.70	176.02	0.23	-3.10	down
**AP002373.2**	2.54E-02	1.94E-03	0.88	29.17	0.29	3.10	up

**PGF**	2.54E-02	1.94E-03	0.59	210.32	0.19	3.10	up
**PKD1L3**	2.64E-02	2.05E-03	0.57	33.74	0.18	3.08	up
**IRF8**	2.65E-02	2.07E-03	0.58	92.55	0.19	3.08	up
**AC137800.2**	2.69E-02	2.10E-03	0.96	19.31	0.31	3.08	up
**ERFE**	2.72E-02	2.14E-03	1.06	15.05	0.35	3.07	up
**CYP4F2**	2.75E-02	2.19E-03	0.59	580.10	0.19	3.06	up
**FAM90A1**	2.75E-02	2.20E-03	-0.62	24.00	0.20	-3.06	down
**CD48**	2.76E-02	2.21E-03	0.93	48.35	0.30	3.06	up
**MALRD1**	2.77E-02	2.22E-03	-0.56	96.12	0.18	-3.06	down
**SLITRK6**	2.77E-02	2.23E-03	-0.76	209.76	0.25	-3.06	down
**KRT16**	2.85E-02	2.31E-03	-0.69	38646.90	0.23	-3.05	down
**KRT6C**	2.87E-02	2.34E-03	-0.82	3503.59	0.27	-3.04	down
**IGLL5**	3.01E-02	2.49E-03	1.89	14.53	0.63	3.02	up
**CCDC105**	3.03E-02	2.52E-03	-0.98	15.20	0.32	-3.02	down
**NKAIN3**	3.06E-02	2.57E-03	-0.63	41.89	0.21	-3.02	down
**RRAD**	3.06E-02	2.56E-03	0.76	59.37	0.25	3.02	up
**ST13P15**	3.10E-02	2.62E-03	-0.81	18.81	0.27	-3.01	down
**IGLC3**	3.12E-02	2.64E-03	1.73	24.64	0.58	3.01	up
**NPBWR1**	3.16E-02	2.68E-03	-0.68	104.28	0.23	-3.00	down
**CCM2L**	3.16E-02	2.69E-03	-0.52	36.66	0.17	-3.00	down
**CES1**	3.19E-02	2.72E-03	0.60	43.13	0.20	3.00	up
**RBMS1P1**	3.22E-02	2.76E-03	0.64	36.38	0.21	2.99	up
**KDELC1P1**	3.22E-02	2.77E-03	-0.64	52.85	0.21	-2.99	down
**NANOGP2**	3.28E-02	2.84E-03	0.74	16.67	0.25	2.98	up
**STS**	3.33E-02	2.89E-03	0.54	253.13	0.18	2.98	up
**IGLV2-14**	3.33E-02	2.89E-03	2.40	13.89	0.81	2.98	up
**CD72**	3.35E-02	2.92E-03	0.52	58.08	0.17	2.98	up
**SERPINB3**	3.37E-02	2.94E-03	0.82	2681.72	0.28	2.97	up
**WDFY4**	3.38E-02	2.95E-03	0.64	283.73	0.22	2.97	up
**COL4A4**	3.42E-02	3.01E-03	0.56	88.35	0.19	2.97	up
**CDHR5**	3.43E-02	3.02E-03	-0.61	151.30	0.21	-2.97	down
**FCGR3B**	3.49E-02	3.09E-03	0.95	31.26	0.32	2.96	up
**AC098582.1**	3.58E-02	3.21E-03	0.52	83.34	0.18	2.95	up
**HEATR9**	3.59E-02	3.23E-03	0.91	19.02	0.31	2.94	up
**AP000295.1**	3.69E-02	3.35E-03	1.30	36.06	0.44	2.93	up
**CRLF2**	3.73E-02	3.42E-03	0.89	16.63	0.30	2.93	up
**KRT8P31**	3.84E-02	3.55E-03	0.53	38.15	0.18	2.92	up
**ALPK2**	3.94E-02	3.70E-03	0.74	60.11	0.26	2.90	up
**UNC5D**	4.00E-02	3.77E-03	-0.65	69.59	0.23	-2.90	down
**SLC27A2**	4.00E-02	3.78E-03	0.51	184.44	0.17	2.90	up
**MYO3A**	4.06E-02	3.86E-03	-0.94	42.27	0.32	-2.89	down
**GDA**	4.06E-02	3.87E-03	0.51	454.55	0.18	2.89	up
**STC2**	4.07E-02	3.89E-03	0.75	33.92	0.26	2.89	up
**REC114**	4.08E-02	3.91E-03	0.51	49.12	0.18	2.89	up

**IGHM**	4.21E-02	4.07E-03	1.39	12.67	0.48	2.87	up
**FPR1**	4.23E-02	4.10E-03	0.54	56.05	0.19	2.87	up
**BMP5**	4.23E-02	4.11E-03	-0.67	29.86	0.23	-2.87	down
**CARMIL2**	4.23E-02	4.12E-03	0.77	35.37	0.27	2.87	up
**PCDHB2**	4.25E-02	4.14E-03	-0.51	91.87	0.18	-2.87	down
**PEG3**	4.25E-02	4.14E-03	-0.53	471.83	0.18	-2.87	down
**VWCE**	4.39E-02	4.33E-03	0.56	29.20	0.20	2.85	up
**GPR151**	4.39E-02	4.34E-03	0.76	15.31	0.27	2.85	up
**RALYL**	4.44E-02	4.41E-03	-0.79	22.15	0.28	-2.85	down
**IGKV3-15**	4.53E-02	4.55E-03	1.44	12.18	0.51	2.84	up
**SNURF**	4.55E-02	4.57E-03	-0.57	44.53	0.20	-2.84	down
**IL4I1**	4.68E-02	4.75E-03	0.84	25.42	0.30	2.82	up
**GPR171**	4.69E-02	4.78E-03	0.71	24.53	0.25	2.82	up
**THEMIS**	4.72E-02	4.82E-03	0.84	40.16	0.30	2.82	up
**CDA**	4.80E-02	4.93E-03	0.51	247.71	0.18	2.81	up
**AC093270.1**	4.81E-02	4.95E-03	-0.86	23.72	0.30	-2.81	down
**FGF18**	4.81E-02	4.96E-03	-0.84	347.15	0.30	-2.81	down
**RPS19P3**	4.81E-02	4.97E-03	0.76	15.62	0.27	2.81	up
**PADI4**	4.84E-02	5.00E-03	0.67	103.76	0.24	2.81	up
**RPS4XP14**	4.89E-02	5.08E-03	0.58	30.00	0.21	2.80	up
**KCNU1**	4.91E-02	5.12E-03	-0.56	121.91	0.20	-2.80	down
**NAGS**	4.92E-02	5.15E-03	0.56	47.44	0.20	2.80	up
**SLURP1**	4.93E-02	5.16E-03	0.55	403.62	0.20	2.80	up
**C8orf74**	4.98E-02	5.21E-03	0.51	99.71	0.18	2.79	up

Ps.The occipital group was used as the reference group
